# Review of the genera *Anelaphinis* Kolbe, 1892 and *Atrichelaphinis* Kraatz, 1898 (Coleoptera, Scarabaeidae, Cetoniinae)

**DOI:** 10.3897/zookeys.482.8343

**Published:** 2015-02-16

**Authors:** Sébastien Rojkoff, Renzo Perissinotto

**Affiliations:** 1 25 avenue Jean Jaurès F-69007 Lyon, France; 2 Department of Zoology, Nelson Mandela Metropolitan University, PO Box 77000, Port Elizabeth 6031, South Africa

**Keywords:** Africa, *Cetonia dominula*, *Anelaphinis*, *Atrichelaphinis*, *Heterelaphinis*, *Megalleucosma*, *Niphetophora*, synonymy, new taxa

## Abstract

New material collected recently throughout the Afrotropical region has led to a major reassessment of taxa within the genera *Anelaphinis* Kolbe, 1892, *Atrichelaphinis* Kraatz, 1898 and other closely related genera. As a result, the name *Megalleucosma* Antoine, 1989 is here synonymised with *Anelaphinis* and a lectotype is designated for the type species, *Cetonia dominula* Harold, 1879. The genus *Atrichelaphinis* is redefined and a new subgenus, Atrichelaphinis (Eugeaphinis), is proposed for *Elaphinis simillima* Ancey, 1883, *Elaphinis vermiculata* Fairmaire, 1894, *Niphetophora rhodesiana* Péringuey, 1907, *Atrichelaphinis deplanata* Moser, 1907 (with *Anelaphinis kwangensis* Burgeon, 1931 as junior synonym) and *Anelaphinis sternalis* Moser, 1914. Additionally, three new species and one new subspecies are recognised and described in this new subgenus: Atrichelaphinis (Eugeaphinis) bomboesbergica **sp. n.** from South Africa; Atrichelaphinis (Eugeaphinis) bjornstadi **sp. n.** from Tanzania; Atrichelaphinis (Eugeaphinis) garnieri **sp. n.** from south–east Africa (Tanzania, Zimbabwe); and Atrichelaphinis (Eugeaphinis) deplanata minettii **ssp. n.** from central Africa (Malawi, Mozambique, Congo-Kinshasa, Congo-Brazzaville, South Africa, Rwanda, Zambia, Zimbabwe). The genus *Atrichelaphinis* is compared to its closest relatives and two separate keys are proposed, one for *Atrichelaphinis* and one for the sub-Saharan genera exhibiting completely or partially fused parameres.

## Introduction

A number of new taxa closely associated with the genus *Anelaphinis* Kolbe, 1892 have recently been reported through intensified work in a number of Afrotropical countries. An attempt to integrate these into existing generic groups has led to a fresh analysis of the type specimens of the species previously included in this genus. This has revealed a state of relative confusion and great uncertainty about the allocation of previously described species to a number of closely related genera that have proliferated during the past century. This, combined with the realization that both genera *Anelaphinis* and *Atrichelaphinis* Kraatz, 1898 have effectively not been subject to any substantial revision since their original description (cf. Holm and Marais 1992), has prompted a full investigation of their current state and taxonomic development.

The two genera *Atrichelaphinis* and *Anelaphinis* exhibit simplesiomorphic similarities between each other and with a number of other closely related genera (Holm and Marais 1992). The key character between the two has generally been considered to be the number of protibial denticles, with *Atrichelaphinis* showing three denticles, with the anterior two extremely approximated, while *Anelaphinis* exhibits one to three denticles poorly approximated. The two genera, on the other hand, share a common aedeagal structure, exhibiting completely fused parameres. In the view of the complexity highlighted above, these characters are now insufficient to allow the unequivocal allocation of several species within either of the two genera. It is, therefore, necessary to revise the taxonomic structure of these and other related genera, by incorporating a new, expanded set of diagnostic characters that can assist with the fine-scale resolution of the species group in question.

## Methods

The description of morphological characters follows the terminology used in Holm and Marais (1992). The length of each specimen excludes head and pygidium, and was measured from the anterior margin of the pronotum to the apex of the elytra. Specimen width represents the maximum width of the elytra, at the level of the humeral callus. Photos of the specimens selected for illustrations were taken using a Nikon D3200 camera fitted with a Nikkor 105 mm objective and Kenko macrotubes. Alternatively, a Canon EOS 550D fitted with a Canon EF 100 mm 1/28 Macro USM lens and a Canon Power Shot S45, combined with a Leica MZ16 dissecting microscope, were used to obtain finer details. Photos were processed with photo stacking technique, using Combine ZP (free software by Alan Hadley, http://www.hadleyweb.pwp.blueyonder.co.uk).

Collection abbreviations used within the text are as follows:

BMNH The Natural History Museum, London, UK

CCEC Center for the Curation and Study of Collections, Lyon, France

IRSN Belgian Royal Institute of Natural Sciences, Bruxelles, Belgium

ISAM Iziko South African Museum, Cape Town, South Africa

MNHN National Museum of Natural History, Paris, France

MNHU Natural Sciences Museum of the Humboldt University, Berlin, Germany

MRAC Royal Museum for Central Africa, Tervuren, Belgium

NMKE National Museum of Kenya, Nairobi, Kenya

PCAB Private Collection Anders Bjørnstad, Skien, Norway

PCDC Private Collection Didier Camiade, Sallespisse, France

PCJT Private Collection Julien Touroult, Soyaux, France

PCPA Private Collection Philippe Antoine, Roubaix, France

PCRM Private Collection Robert Minetti, La Ciotat, France

PCRP Private Collection R Perissinotto & L Clennell, Port Elizabeth, South Africa

PCSR Private Collection Sébastien Rojkoff, Lyon, France

PCTB Private collection Thierry Bouyer, Chênée, Belgium

PCTG Private Collection Thierry Garnier, Montpellier, France

TMSA Ditsong National Museum of Natural History (formerly Transvaal Museum), Pretoria, South Africa

## Taxonomic account

### Genus *Anelaphinis* Kolbe, 1892

**Figure 1.**
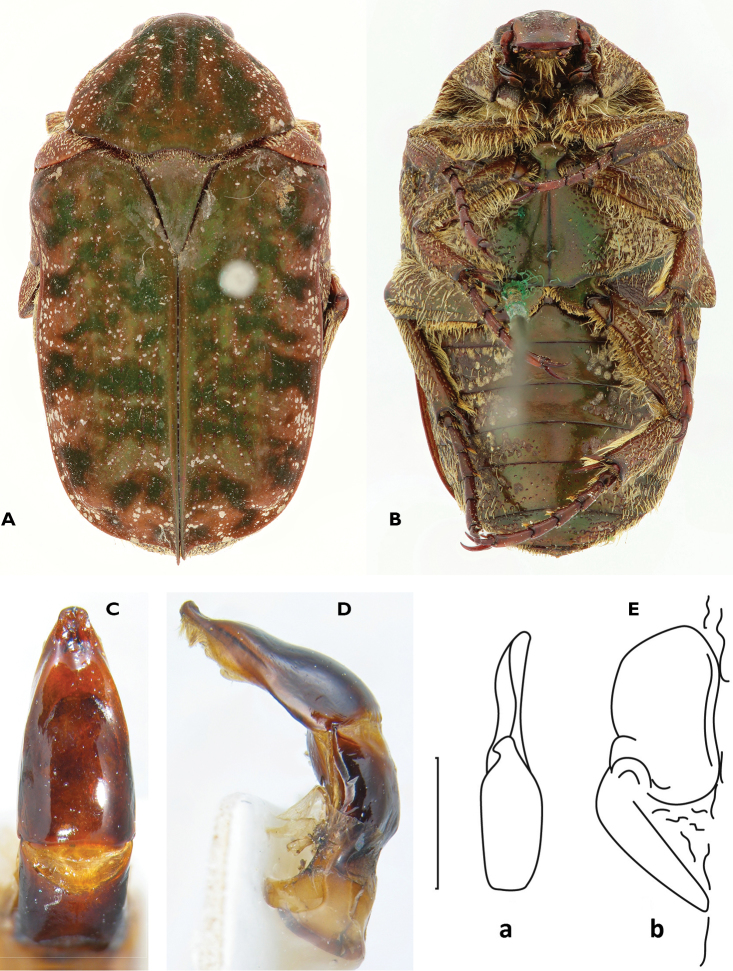
*Anelaphinis dominula* (Harold, 1879), Holotype (MNHU). **A** Dorsal view **B** ventral view **C** parameres in dorsal view **D** parameres in lateral view **E** sclerite of internal sac (**a** dorsal view; **b** lateral view) (Scale bar = 1 mm).

After clarifying an erroneous identification of *Cetonia dominula* Harold, 1879, contained in Kraatz (1880: 172–173; 1892: 415), Kolbe (1892a: 135–136) created the genus *Anelaphinis*, designating *Cetonia dominula* as its type species. Upon a closer analysis of the four syntypes deposited at MNHU, a lectotype is here designated. This is a male specimen (Figure 1) carrying the following eight labels: 1) (blue-grey colour) "dominula Harold, Angola or."; 2) (red-orange colour) "type"; 3) (white colour) "60113"; 4) (white colour) "Anelaphinis dominula Harold type ♂"; 5) (yellow colour) "Zool. Mus. Berlin"; 6) (blue colour) "Hist. Coll. (Coleoptera)/Nr. 60113/Cetonia dominula Harold*/Malange Homeyer & Schütt/ Zool. Mus. Berlin"; 7) (red colour) "Syntypus Anelaphinis dominula (Harold 1879) labelled by MNHUB 2012"; 8) (white colour with red margin) present designation "Lectotype *Anelaphinis dominula* (Harold, 1879) S. Rojkoff 2012". The other three syntypes are labelled as "Paralectotypes". All syntypes match perfectly the description of Harold (1879: 77). Kolbe (1892a) did not provide a detailed description of the genus, but briefly compared it to the genera *Macrelaphinis* Kraatz, 1880, *Niphobleta* Kraatz, 1880 and even to the Asian *Protaetia* Burmeister, 1842. Shortly after, he described *Eucosma breviceps* (Kolbe 1892b: 253).

A study of the type series of *Anelaphinis dominula* reveals the presence of all characters used to define *Megalleucosma* Antoine, 1989 (type species: *Eucosma breviceps* Kolbe, 1892), especially in the structure of the aedeagus and the sclerite of the internal sac (Figures 1C–E). Consequently, *Megalleucosma* Antoine, 1989 is here considered as a junior synonym of *Anelaphinis* Kolbe, 1892 (syn. n.). *Anelaphinis dominula* is definitely not a synonym of any of the species considered by Antoine (1989), as clearly shown in his comparative aedeagal study.

It is not known what prompted Kolbe (1892b: 253) to describe *Eucosma breviceps* from Barombi station, Cameroun, as belonging to another genus only a few months after the description of the genus *Anelaphinis*, despite their obvious generic similarities. De Lisle (1947: 38-39) was among the few to have correctly used the genus *Anelaphinis* in his description of *Anelaphinis vaillanti*. Schauer too, was able to assign members of the genus accurately, as evidenced by his determinations of the MNHU material. Ruter (1960: 437) recognised the synonymy of *Anelaphinis vaillanti* with *Eucosma breviceps*, but regarded it as a member of *Alleucosma*. He acknowledged the complexity of this group of genera and the difficulty of placing species correctly within them, but did not subscribe to De Lisle’s (1947) proposal.

Among the other species usually placed within *Anelaphinis* are: *Elaphinis simillima* Ancey, 1883; *Elaphinis vermiculata* Fairmaire, 1894; *Niphetophora rhodesiana* Péringuey, 1907; *Atrichelaphinis deplanata* Moser, 1907; *Atrichelaphinis sternalis* Moser, 1914; and *Atrichelaphinis kwangensis* Burgeon, 1931. All have in common an aedeagus with parameres completely fused and without sclerite in the internal sac. Eventually, this character has been regarded as key to the diagnosis of the genus *Anelaphinis* (e.g. Holm and Marais 1992). With the exception of Antoine (1989), there has been no attempt to take into account other characters of the aedeagus in the taxonomy of this and other closely related genera. This includes Moser (1914: 606), who described *Atrichelaphinis simillima* on the basis of a male specimen only and then compared it to *Cetonia dominula*; as well as Schenkling (1921: 306), who reported *Cetonia dominula* from Angola and Ethiopia. Schenkling’s position, probably taken on the basis of the work of Schoch (1896: 384), was promptly followed by Burgeon (1931: 219, 221) who described *Atrichelaphinis collarti* and *Atrichelaphinis kwangensis* with drawings of their aedeagus, and Burgeon (1934: 260) reporting "*Anelaphinis* apud *dominula*" from Elisabethville (Congo-Kinshasa) without any other specification (see also Ferreira 1965: 1207; Marais and Holm 1992: 7 and 47; Krajcik 1998: 50). The classification into different genera has largely relied on external morphological characters and on the colour pattern of the dorsal habitus. Only recently, Antoine (1987: 464; 1988: 48; 1989a: 31; 1989b: 245; 1991: 2) and Antoine and Holm (1993: 101–102) were able to clarify the taxonomic position of most of these closely related genera, namely: *Alleucosma* Schenkling, 1921; Alleucosma (Eoalleucosma) Antoine, 1989; *Analleucosma* Antoine, 1989; *Aphelinis* Antoine, 1987; *Heteralleucosma* Antoine, 1989; *Molynoptera* Kraatz, 1897; *Molinopteroides* Antoine, 1989; *Niphetophora* Kraatz, 1883; *Paralleucosma* Antoine, 1989; *Paranelaphinis* Antoine, 1988; *Phaneresthes* Kraatz, 1894 and *Pseudalleucosma* Antoine, 1989.

### Summary of the current taxonomic composition of the genus *Anelaphinis* Kolbe, 1892

*Anelaphinis* Kolbe, 1892

*Alleucosma* Schenkling, 1921, *partim*.

*Megalleucosma* Antoine, 1989: 248, 265, **syn. n.**

Type species: *Cetonia dominula* Harold, 1879

*Anelaphinis allardi* (Ruter, 1978)

*Eucosma allardi* Ruter, 1978

*Megalleucosma allardi* (Ruter) Antoine, 1989: 269

*Anelaphinis bourgoini* (Burgeon, 1932)

*Alleucosma bourgoini* Burgeon, 1932

*Megalleucosma bourgoini* (Burgeon) Antoine, 1989: 271

*Anelaphinis breviceps* (Kolbe, 1892)

*Eucosma breviceps* Kolbe, 1892: 253

*Alleucosma breviceps* (Kolbe, 1892)

*Anelaphinis vaillanti* De Lisle, 1947: 38

*Megalleucosma breviceps* (Kolbe) Antoine, 1989: 271

*Anelaphinis dominula* (Harold, 1879)

*Cetonia dominula* Harold, 1879

*Anelaphinis maritima* (Moser, 1914)

*Eucosma maritima* Moser, 1914

*Alleucosma maritima* Moser, 1914

*Alleucosma maritimi* Schein, 1956: 194

*Anelaphinis pauliani* (Antoine, 1989)

*Megalleucosma pauliani* Antoine, 1989: 268

*Anelaphinis similis* (Antoine, 1989)

*Megalleucosma similis* Antoine, 1989: 270

### Genus *Atrichelaphinis* Kraatz, 1898

This genus was erected by Kraatz (1898: 220), in order to accommodate species close to, but different from those included in *Elaphinis* by Burmeister (1842). Without designating a type species, Kraatz (1898) included in *Atrichelaphnis* three species, *Cetonia irrorata* Fabricius, 1798, *Cetonia tigrina* Olivier, 1789 and *Elaphinis vermiculata* Fairmaire, 1894, mainly on the basis of their sharing a bilobed "ligula" and the shape of the metatibial spur. Kraatz was familiar with the genus *Micrelaphinis* Schoch, 1896, having described in 1896 varieties of *Micrelaphinis mutabilis* Schoch, 1895, but failed to recognise *Cetonia irrorata* as part of this genus, despite the diagnostic shape of its clypeus. This was only rectified later by Péringuey (1907: 339). Marais and Holm (1989) redefined the taxonomic position of the genus *Elaphinis* Burmeister, 1842 and its relationships with *Micrelaphinis* Schoch, 1894. They (re-)transferred *Elaphinis vermiculata* Fairmaire, 1894 to *Atrichelaphinis s. l.*, on the basis of the fused aedeagal parameres and, while highlighting the need to undertake a revision of the genus, they ignored the original work of Kraatz (1898), who had already placed *Elaphinis vermiculata* in *Atrichelaphinis*. In their catalogue (Marais and Holm 1992: 11), *Cetonia tigrina* Olivier, 1789 was designated as type species for the genus, which at that stage comprised four species: *Atrichelaphinis deplanata* Moser, 1907 (synonym: *Anelaphinis kwangensis* Burgeon, 1931; Antoine 1988: 48); *Cetonia nigropunctulata* Péringuey, 1896; *Elaphinis quadripunctata* Lansberge, 1882; and *Cetonia tigrina* Olivier, 1789 (synonym: *Cetonia furvata* Fabricius, 1798). No further elaboration on the genus was provided in Holm and Marais (1992: 195), where only the two South African species were considered, *Atrichelaphinis nigropunctulata* and *Atrichelaphinis tigrina*.

Following this, Antoine (2002: 182–186) redefined *Atrichelaphinis s. s.* as composed of two species, *Atrichelaphinis tigrina* and *Atrichelaphinis nigropunctulata*. He created the new sub-genus *Heterelaphinis*, with *Cetonia quadripunctata* Lansberge, 1882 as type species and including *Leptothyrea sexualis* Schein, 1956, as well as the newly described Atrichelaphinis (Heterelaphinis) nigra Antoine, 2002. Simultaneously, he transferred *Atrichelaphinis deplanata* and *Elaphinis vermiculata* Fairmaire, 1894 to the genus *Anelaphinis* on the basis of their pronotal shape.

The consequences of the confusion created with the genus *Atrichelaphinis* and with the species previously included in *Anelaphinis* are that currently their taxonomic position remains unclear (with the exception of *Anelaphinis dominula*) and badly in need of revision. Only two options appear to be viable at the moment: 1) including them in an existing genus; or 2) erecting a new taxonomic entity especially for them. Upon completing an extensive analysis of many specimens for each species, the following key diagnostic characters are here selected for the new taxonomic order proposed in the section below.

Diagnostic characters: - ventral surface, with extensive scale-type hair cover;
- clypeus, ratio of length/width;
- anterior clypeal margin, with inflexions and/or ridges;
- antennal club, longer in male (sexual dimorphism);
- pronotal shape, of octogonal type;
- pronotal structure, surface more or less bulbous/tectiform, tuberculate or without any modification at middle of the frontal margin, posterior margin more or less emarginate in front of the scutellum;
- mesosternal apophysis, transverse;
- elytra, tricostate;
- protibiae, bi- or tridentate with variable denticle positions;
- meso- and metatibiae, exhibiting external carina;
- terminal spurs of metatibiae showing sexual dimorphism;
- parameres, completely fused and showing apical expansions, apex more or less curved downwards, usually with small median incision/sinuosity;
- female genitalia.

As suggested by Ruter (1960), the main diagnostic character for the separation of the "*Elaphinis*-type" genera is the aedeagus. As all the above mentioned species exhibit completely fused parameres, with internal sac lacking the sclerite, and most features generally associated with *Atrichelaphis*, this is the only suitable genus for this species group. No other genus matches these characteristics closely enough to warrant consideration. A minor problem is that not all the characteristics mentioned above are perfectly suitable for the incorporation of both subgenera, as defined by Antoine (2002). Nevertheless, the work of Antoine is here confirmed through new diagnostic characters and supported, as it provides a valuable proposal for the way forward. Some important remarks are, however, necessary at this stage.

Firstly, Antoine (2002) defined *Atrichelaphinis* mainly through a pronotal character, describing its posterior border straight or slightly concave in front of the scutellum. This is not a clear-cut character and could potentially generate misunderstandings. The study of several hundred specimens of the two species belonging to *Atrichelaphinis s. s.* shows a posterior pronotal border with a straight or slightly concave median part, while on both sides the border is largely curved down to the rounded posterior angles. The edge of the posterior angles is in front of the straight median part of the posterior border (in front of the scutellum). This contradicts Antoine’s (2002) statement that "*marge postérieure du pronotum non étirée obliquement en arrière*" and qualifies the posterior border as consisting of three different parts, or bisinuate in shape.

Secondly, Antoine also separated the nominal subgenus from *Heterelaphinis* through the shape of the aedeagal parameres, with apical median protrusion incised or not, the protibias bi- or tridentate and the antennal club, either equal in the two sexes of *Atrichelaphinis s. s.* or longer in the male of *Heterelaphinis*. However, observations undertaken during this study show that the antennal club is always longer in males than in females, in both subgenera, with maximum difference observed in *Heterelaphinis*. To appreciate correctly this character, it is necessary to compare specimens of the same size. This observation is also valid for the four species previously included in *Anelaphinis* mentioned above.

Antoine (2002) separated the species of *Anelaphinis* and *Atrichelaphinis* using as key characters protibiae bi- or tridentate and apex of the parameres reployed or not. However, he omitted another important character: the clypeus, which is transverse and without sexual dimorphism in *Atrichelaphinis* while it is longer than wide in the three species of *Heterelaphinis*.

The ex-*Anelaphinis* species exhibit the main characteristics of the genus *Atrichelaphinis s. l.*, which should be enough not to erect a new genus. There are, however, two features that do not allow a similar, full placement of some species within this genus. These are a transverse clypeus with sexual dismorphism, protibiae bidentate with wide separation between the teeth, in association with completely fused parameres without apical modification and just curved downwards, rather than reployed. To include species exhibiting such characters, we consider as necessary to erect a new subgenus, Atrichelaphinis (Eugeaphinis) subgen. n. The implication of this is that the genus *Atrichelaphinis* and the two recognized subgenera must be redefined. Thus, the classification of Antoine (2002) is completed and modified here below.

### Summary of the current taxonomic composition of the genus *Atrichelaphinis* Kraatz, 1898

Atrichelaphinis (Atrichelaphinis) Kraatz, 1898

*Elaphinis* Péringuey, 1907.

Type species: *Cetonia tigrina* Olivier, 1789

Atrichelaphinis (Atrichelaphinis) tigrina (Olivier, 1789)

*Cetonia tigrina* Olivier, 1789

Cetoninus (Cetonia) tigrina (Olivier) MacLeay, 1838

*Elaphinis tigrina* (Olivier)

*Cetonia furvata* Fabricius, 1798

*Atrichelaphinis furvata* (Fabricius)

*Euryomia furvata* (Fabricius)

Atrichelaphinis (Atrichelaphinis) nigropunctulata (Péringuey, 1896)

*Cetonia nigropunctulata* Péringuey, 1896

*Elaphinis nigropunctulata* (Péringuey)

*Elaphinis nigropunctata* (Péringuey)

Atrichelaphinis (Heterelaphinis) Antoine, 2002

Type species: *Elaphinis quadripunctata* Lansberge, 1882

Atrichelaphinis (Heterelaphinis) quadripunctata (Lansberge, 1882)

*Elaphinis quadripunctata* Lansberge, 1882

*Atrichelaphinis quadripunctata* (Lansberge)

*Cetonia quadripunctata* (Lansberge)

Atrichelaphinis (Heterelaphinis) sexualis (Schein, 1956)

*Leptothyrea sexualis* Schein, 1956

Atrichelaphinis (Heterelaphinis) nigra Antoine, 2002

Atrichelaphinis (Eugeaphinis)**subgen. n.**

*Pseudanelaphinis* Antoine (*in litteris*)

Type species: *Atrichelaphinis deplanata* Moser, 1907

Atrichelaphinis (Eugeaphinis) deplanata deplanata (Moser, 1907)

*Atrichelaphinis deplanata* Moser, 1907

*Atrichelaphinis deplanate* (Moser)

*Anelaphinis deplanata* Moser

*Anelaphinis kwangensis* Burgeon, 1931

*Atrichelaphinis kwangensis* (Burgeon)

Atrichelaphinis (Eugeaphinis) deplanata minettii**subsp. n.**

Atrichelaphinis (Eugeaphinis) rhodesiana (Péringuey, 1907)

*Niphetophora rhodesiana* Péringuey, 1907

*Anelaphinis rhodesiana* (Péringuey)

Atrichelaphinis (Eugeaphinis) bomboesbergica**sp. n.**

Atrichelaphinis (Eugeaphinis) garnieri**sp. n.**

Atrichelaphinis (Eugeaphinis) simillima (Ancey, 1883)

*Elaphinis simillima* Ancey, 1883

*Anelaphinis simillima* (Ancey)

*Atrichelaphinis simillima* Müller, 1939

Atrichelaphinis (Eugeaphinis) sternalis (Moser, 1914)

*Anelaphinis sternalis* Moser, 1914

Atrichelaphinis (Eugeaphinis) vermiculata (Fairmaire, 1894)

*Elaphinis vermiculata* Fairmaire, 1894

*Anelaphinis vermiculata* (Fairmaire)

*Atrichelaphinis vermiculata* (Fairmaire)

Atrichelaphinis (Eugeaphinis) bjornstadi**sp. n.**

### Atrichelaphinis s. l.

Taxon classificationAnimaliaColeopteraScarabaeidae

Kraatz, 1898

#### Generic characters.

**Figure 2.**
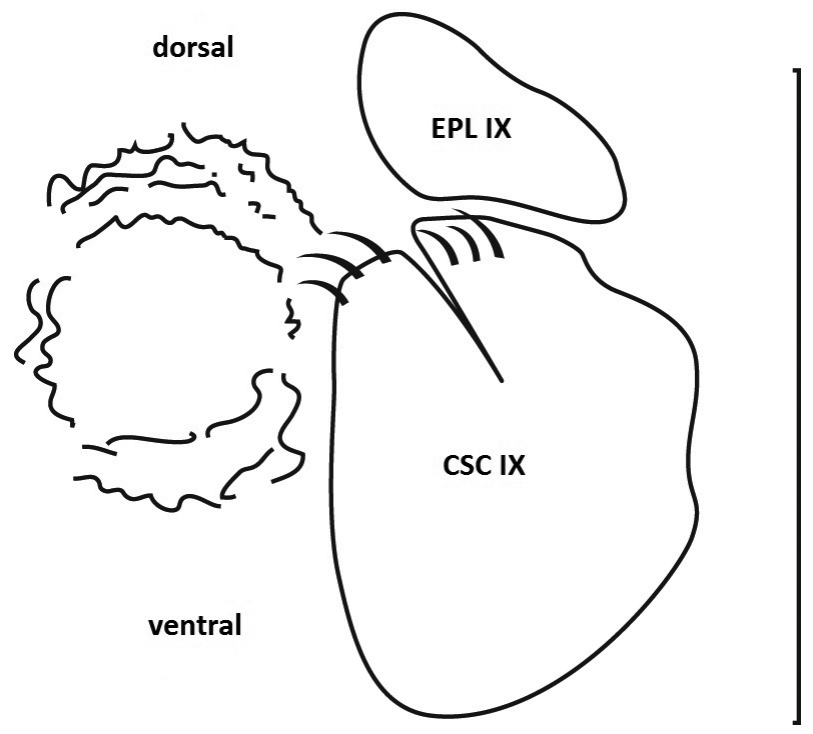
Atrichelaphinis (Eugeaphinis) deplanata (Moser, 1907). Female genitalia (EPL IX: epipleurite IX; CSC IX: coxosubcoxite IX) (Scale bar = 1 mm).

Clypeus rectangular; antennal club longer in male than in female; pronotum sub-octogonal, anterior border convex with or without projection, posterior border largely convex, more or less bisinuate, posterior angles not acute; scutellum longer than wide, apex from more or less acute to slightly dull; elytra tricostate; mesosternal apophysis tansverse; male abdomen concave with a median depression; protibia bi- or tridentate, meso- and metatibias with transverse carina on external side, metatibia widened and thickened at apex; aedeagus with parameres fused, except at apex, internal sac without sclerite; female genitalia (Figure 2) exhibiting regression of tergite and retention of epipleurite IX as dorsopleurite, showing articulation on simplified gonopod, with gonopod composed of coxosubcoxite IX made of partial fusion of coxite and subcoxite.

### Atrichelaphinis (Atrichelaphinis)

Taxon classificationAnimaliaColeopteraScarabaeidae

Kraatz, 1898

Atrichelaphinis Kraatz, 1898; Distant 1911: 266; Schenkling 1921: 304; Marais and Holm 1992: 11.
Elaphinis Péringuey, 1907; Schenkling 1921: 304; Schein 1960: 98.

#### Type species.

*Cetonia tigrina* Olivier, 1789.

#### Subgeneric characters.

Clypeus transverse (almost as long as wide) without sexual dimorphism, bilobed; median part of pronotal posterior border either straight or slightly concave in front of scutellum; protibia tridentate, with two distal denticles very close to each other, proximal tooth sometimes very reduced or as slight sinuosity; metatibial apical spurs not enlarged in female; aedeagus with parameres fused and apically reployed downwards, except sometimes with small incision in downturning apical part (or sinuation when such incision is absent). Two species are currently included in the nominal subgenus.

### Atrichelaphinis (Atrichelaphinis) tigrina

Taxon classificationAnimaliaColeopteraScarabaeidae

(Olivier, 1789)

Figure 3

Cetonia tigrina Olivier, 1789: 91; Gory and Percheron 1833: 272; MacLeay 1838: 46; Boheman 1857: 27; Schenkling 1921: 304; Antoine 1991: 2; Marais and Holm 1992: 11; Holm and Marais 1992: 197; Antoine 2002: 185.
Cetoninus (Cetonia) tigrina (Olivier) MacLeay, 1838: 46.
Elaphinis tigrina (Olivier) Blanchard, 1850: 12; Ancey 1883: 95; Kraatz 1883: 384; Gerstaecker 1884: 46; Fairmaire 1893: 10; Schoch 1895: 108; Kraatz 1895a: 382; Distant 1897: 576; Péringuey 1907: 449; Schenkling 1921: 304; Holm and Marais 1992: 197; Antoine 2002: 185.
Atrichelaphinis tigrina (Olivier) Moser, 1907: 321; Péringuey 1908: 684; Distant 1911: 266; Schenkling 1921: 304; Marais and Holm 1992: 11; Holm and Marais 1992: 197; Antoine 2002: 185.
Atrichelaphinis furvata (Fabricius) Marais & Holm, 1992: 11.
Cetonia furvata Fabricius, 1798: 130; Thunberg 1818: 420; Boheman 1857: 27; Schenkling 1921: 304; Antoine 1991: 2; Marais and Holm 1992: 11; Holm and Marais 1992: 197; Antoine 2002: 185.
Elaphinis furvata (Fabricius) Burmeister, 1842: 597; Blanchard 1850: 12; Boheman 1857: 27; Harold 1878: 213; Fairmaire 1887: 129; Schenkling 1921: 304; Holm and Marais 1992: 197; Antoine 2002: 185.
Euryomia furvata (Fabricius) Redtenbacher, 1868: 81; Schenkling 1921: 304; Antoine 2002: 185.

#### Type specimen.

Holotype unknown.

#### Redescription

**(n > 100).** Size: length ♂, 8.6–15.2 mm; ♀, 9.6–15.0 mm; width ♂, 5.0–8.8 mm; ♀, 5.3–9.1 mm.

*Body.* Dorsum orange-brown, matt with black marks well defined and more or less developed, especially on head and pronotum; often with white tomentose spots on pronotum, scutellum and exceptionally on elytra; setae short on vertex, pronotum (mainly on lateral side) and elytral base, extremely short and barely visible on clypeus, elytra and pronotal disc.

*Head.* Clypeus almost as long as wide, bilobed and upturned on anterior margin, punctures deep, with setigerous punctures becoming confluent laterally and in front.

*Pronotum.* Angles round, postero-lateral ones sometimes vanishing; posterior margin straight to concave in front of scutellum; with reborded lateral margins.

*Scutellum.* Variably marked with black markings and white tomentose spots; setae barely noticeable, mainly at margins.

*Elytra.* Disc exhibiting three pairs of single to double geminate striae, with first two usually complete, third more or less complete; odd costae convex; sutural apex from blunt to protruding.

*Pygidium.* Black with some light to dark brown areas; lunulate setigerous punctures, sometimes forming a complete ring on the surface but near apex forming more or less horizontal and confluent lines; apex marginated.

*Underside.* Shiny, black with more or less developed brown areas and white tomentose spots on metasternum and abdomen; setae long, scattered and shorter on mesepimera and abdominal sternites; mesosternal apophysis transverse, sometimes very large, compressed between mesocoxae, usually flat, sometimes showing relief, covered with setae, but not protruding in front of them; metasternum laterally strongly sculptured with wrinkles, median part glabrous and smooth, with longitudinal mediam line; abdomen more densely sculptured laterally with horseshoe punctures; concave in males, sometimes with slight groove, convex in females.

*Legs.* Long setae particularly dense on femora and tibiae; metatibiae and metafemora thickened, without tomentum; metatibial spur thin and pointed in male, slightly enlarged and blunt in female.

*Aedeagus.* Parameres subparallel in their apical half, then enlarged; lateral apical angle showing more or less developed hook-like protrusion; downturned part of apex showing incision at middle.

#### Remarks.

One female from the MNHU (Coll. L.W. Schaufuss, labeled "Cap b. Sp.") exhibits protibiae bidentate, with teeth widely separated. Other female specimens have been observed with the same feature, but no males. This seems to be exceptional and possibly due to wearing during the fossorial action required to lay eggs underground.The species is mainly distributed in the eastern part of South Africa, reaching the Western Cape Province along the southern coastline. There are also occasional reports from Zimbabwe and southern Mozambique (Holm and Marais 1992). This is a typical flower and fruit feeder that has been observed on a large variety of plants, from grasses to large trees.

**Figure 3.**
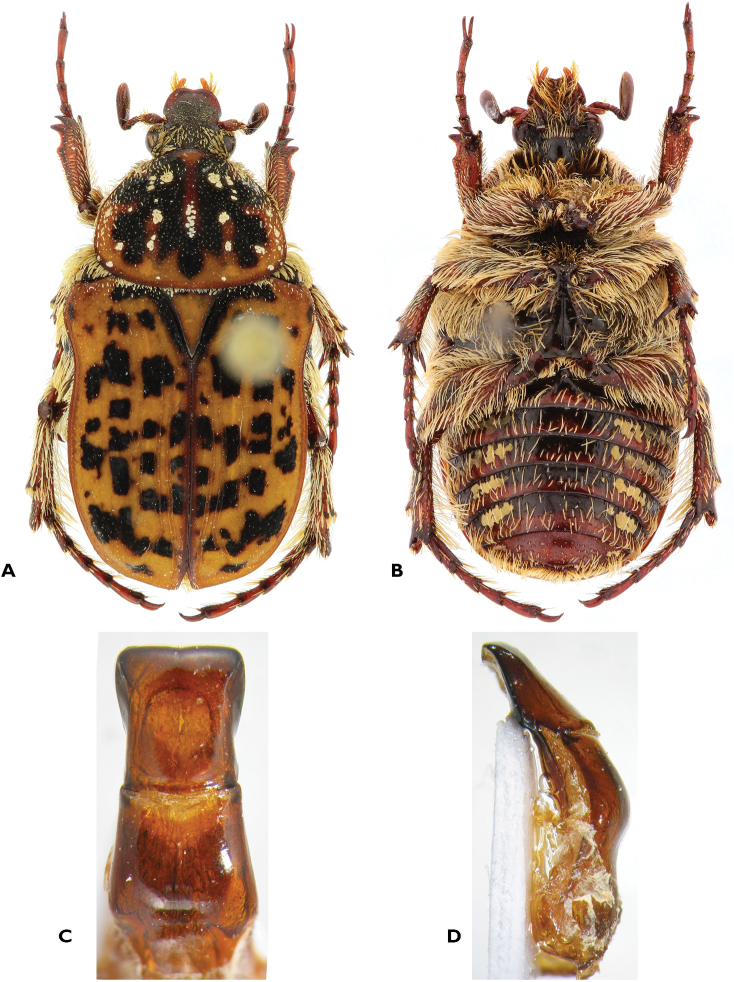
Atrichelaphinis (Atrichelaphinis) tigrina (Olivier, 1789), male, South Africa, Western Cape (PCSR). **A** Dorsal view **B** ventral view **C** parameres in dorsal view **D** parameres in lateral view.

### Atrichelaphinis (Atrichelaphinis) nigropunctulata

Taxon classificationAnimaliaColeopteraScarabaeidae

(Péringuey, 1896)

Figure 4

Cetonia nigropunctulata Péringuey, 1896: 163; Schenkling 1921: 304; Antoine 1991: 2; Marais and Holm 1992: 11; Holm and Marais 1992: 196; Antoine 2002: 185.
Elaphinis nigropunctulata (Péringuey) Péringuey, 1907: 448; Antoine 2002: 185.
Elaphinis nigropunctata (Péringuey) Distant, 1897: 576; Schenkling 1921: 304.
Atrichelaphinis nigropunctulata (Péringuey) Moser, 1907: 321; Distant 1911: 266; Schenkling 1921: 304; Schein 1960: 98; Marais and Holm 1992: 11; Holm and Marais 1992: 196; Krajcik 1998: 50; Antoine 2002: 185.

#### Type specimen.

Holotype in ISAM.

#### Redescription

**(n > 30).** Size: length ♂, 12.8–15.2 mm; ♀, 13.1–14.8 mm; width ♂, 7.7–8.8 mm; ♀, 7.7–8.8 mm.

*Body.* Orange with black markings on pronotum, scutellum and elytra, sometimes very reduced; occasionally showing some isolated small spots of white tomentum on pronotum, pygidium and venter; pilosity occasional and restricted to head.

*Head.* With vertex and lateral part of the frons black, clypeus slightly transverse, bilobed at apex, with anterior margin reborded and lobes slightly upturned. Sculpture deep, simple, becoming confluent in front, laterally and on frons; antennae darker.

*Pronotum.* With angles rounded, lateral margins almost entirely reborded except near posterior angles, lateral angles always marked, posterior part of lateral margin concave; posterior margin concave in front of scutellum, then laterally very convex; sculpture usually weak on disc, generally denser and deeper laterally.

*Scutellum.* Acute, grooved laterally; punctuation limited to anterior angles.

*Elytra.* Sculpture very scattered, disc with two pairs of geminate striae, usually consisting of virtually complete single lines, sometimes merged with horseshoe sculpture, lateral sculpture present or not, series of deep and large points along lateral margin always present; sutural apex from blunt to slightly protruding.

*Pygidium.* Sculpture usually of small points or lines, sometimes of wrinkles and/or horseshoe setigerous punctures; posterior margin slightly reborded; occasionally covered with short setae, particularly around margins.

*Underside.* Black except metepisternum, lateral parts of metacoxae, metasternum and sternites; mesepimeron black or orange; mesosternal apophysis orange with black sides, transverse, compressed between mesocoxae and not protruding; moderately covered with setae, except on abdominal sternites; metasternum with wrinkles and long pilosity laterally, grooved in the middle and poorly sculpted to smooth; abdomen poorly sculpted with setigerous horseshoe punctures, setae short; concave to grooved in males, convex in female.

*Legs.* Metafemora and metatibia enlarged apically, spurs not dilated in either sex; moderately covered with setae, particularly around base; metatibial spur thin and pointed in male, thin and less acute to sligthly blunt in female.

*Aedeagus.* Parameres almost twice as long as wide; basal half converging in front, apical divergent; lateral apical angles showing fairly developed hook.

#### Remarks.

The distribution of this species is restricted to the mountainous northeast part of South Africa. Some specimens could be confused superficially with some forms of *Atrichelaphinis tigrina*, however they can be separated through analysis of the dorsal sculpture, shape of the pygidium and aedeagus. The species is most frequently found feeding on *Protea* spp. flowers.

**Figure 4.**
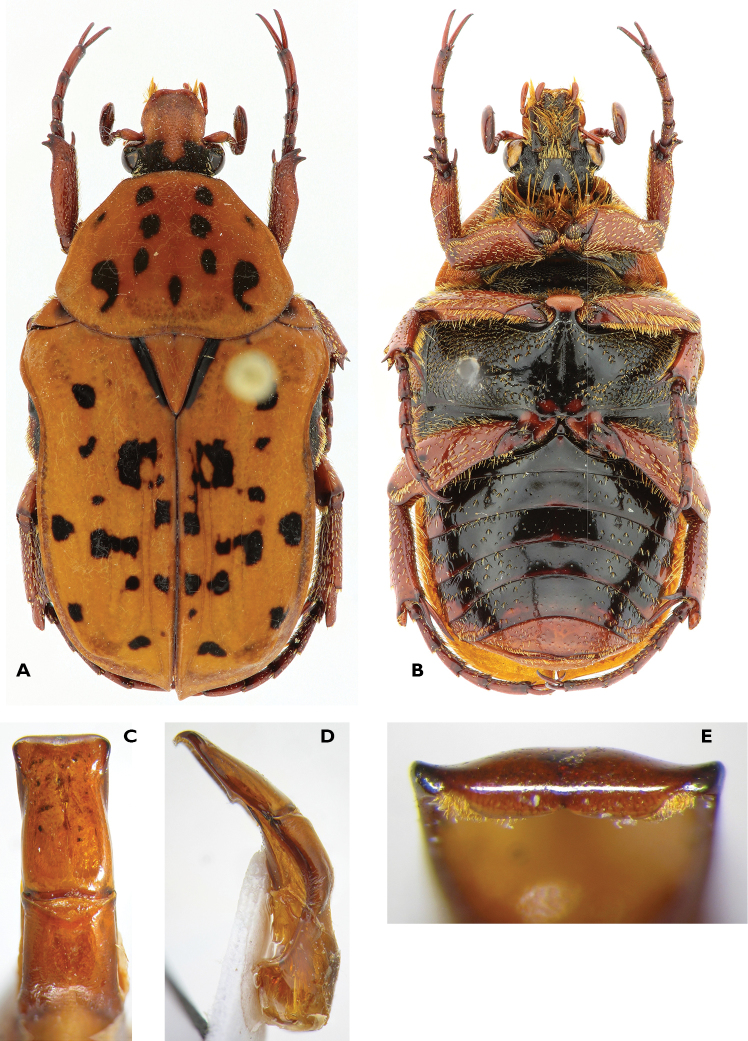
Atrichelaphinis (Atrichelaphinis) nigropunctulata (Péringuey, 1896) male, South Africa, Barberton (PCSR). **A** Dorsal view **B** ventral view **C** parameres in dorsal view **D** parameres in lateral view **E** apex of the parameres.

### Atrichelaphinis (Heterelaphinis)

Taxon classificationAnimaliaColeopteraScarabaeidae

Antoine, 2002

#### Type species.

*Elaphinis quadripunctata* Lansberge, 1882

#### Subgeneric characters.

Clypeus longer than wide with the apex slightly sinuous; anterior pronotal border tuberculate or tectiform, but minimally so in female; protibiae bidentate, with teeth normally separated; metatibial apical spurs strongly enlarged (spatuliform) in female; parameres of aedeagus fused with apex modified (in dorsal view), with protrusion in the middle deeply incised or not, apex laterally modified or not. Three species are currently included in this subgenus.

### Atrichelaphinis (Heterelaphinis) quadripunctata

Taxon classificationAnimaliaColeopteraScarabaeidae

(Lansberge, 1882)

Figures 5 and 6

Elaphinis quadripunctata Lansberge, 1882: 24; Ritsema 1888: 225; Kolbe 1897: 180; Antoine 1991: 2; Marais and Holm 1992: 11.
Atrichelaphinis quadripunctata (Lansberge) Marais & Holm, 1989: 30; Marais and Holm 1992: 11; Krajcik 1998: 50.
Cetonia quadripunctata (Lansberge) Antoine, 2002: 185.

#### Type specimen.

**Figure 5.**
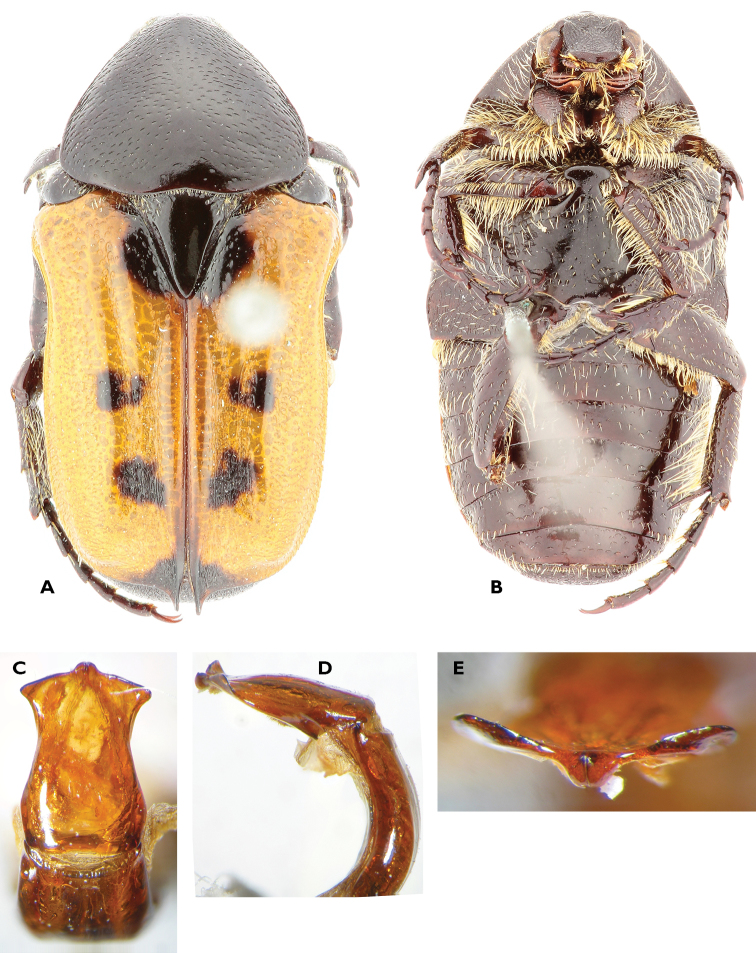
Atrichelaphinis (Heterelaphinis) quadripunctata (Lansberge, 1882), lectotype male, Somalia (MNHN). **A** Dorsal view **B** ventral view **C** parameres in dorsal view **D** parameres in lateral view **E** apex of parameres.

Marais and Holm (1992) mentioned two paralectotypes: one in the BMNH collections and one in the MNHN. The male specimen housed in the MNHN shows the following labels: "Somali, Ouarsangueli, Revoil 1881, Museum Paris/1598 81"; and "Lectotype, Elaphinis quadripunctata van Lansberge, Ph. Antoine det 88". There is, however no reference to this designation in the publications of Antoine (1991, 2002), apart from a mention of the lectotype in the legend to Figure 21 of Antoine (2002). Consequently, in order to settle the status of the species, the male illustrated in Figure 5 is here designated as Lectotype and a new label is added to the two described above, reading: "Lectotype, Elaphinis quadripunctata van Lansberge, Rojkoff & Perissinotto 2014". Four other specimens, identified as *Elaphinis quadripunctata* by Antoine in 1994, were found in the MNHN collections. Two females have the same label as the lectotype and are here designated as paralectotypes. The last specimens, one male missing pronotum and head and a female are only labelled "Ex-Musaeo Van Lansberge" and "Museum Paris, ex. Coll. R. Oberthur". It is possible that these specimens belong to the type series, but as this could not be confirmed during this study, they cannot be designated as paralectotypes here.

#### Redescription

**(n = 7).** Size: length LT ♂, 11 mm; width 5.5 mm.

*Head.* Dark brown with blackish areas, strongly sculpted, converging points forming deep striae; clypeus longer than wide, lateral and anterior margins strongly reborded, anterior slightly upturned and very slightly bilobed, lateral margins almost carinate in the basal part, then curved downwards, depressed in the middle and reborded in the apical part as the anterior margin, clypeal disc convex; frons with large striated protuberance between eyes, vertex with few smooth jointed areas between striae in apical part, posterior part only punctate; antennae brown with long clubs (as long as the flagellum in male).

*Pronotum.* Transverse, dark brown with transverse points of sculpture, disc poorly punctate, sculpture becoming more dense and confluent to striae in front and laterally; anterior margin slightly wider than head, medially slightly tuberculate; lateral margins reborded with very smooth lateral angles in posterior third; posterior margin bisinuate (concave in front of scutellum), posterior angles rounded.

*Scutellum.* Dark brown, longer than wide, apex acute, smooth, only a few setigerous points on lateral angles (scale pilosity); laterally grooved.

*Elytra.* Orange with four black markings, one on side of scutellum, one at middle split on each costa (discal and humeral), one on apical quarter near the suture and last on spiny apex; costae convex, smooth with only few points, discal costa incomplete, humeral costa concave to suture with concavity reaching elytral disc; sculpture of small longitudinal lines (near scutellum) and of horseshoe points in anterior half, becoming confluent posteriorly and leading to two formations: 1) laterally (i.e. between humeral costa and lateral margin) transverse lines becoming longer and denser toward apex; 2) longitunal lines between sutural and humeral costae becoming more numerous and strigillate towards apex; few minute and very short setae near apex.

*Pygidium.* Transverse, chestnut brown; sculpture horseshoe-like to annulate points drawing large irregular circles towards apex, some transverse striae along apex; few minute and very short setae.

*Underside.* From dark brown to chestnut brown, sculpture setigerous with long whitish pilosity, not dense except on femora and laterally on sternites 2–5; sculpture sparce, crescent on metasternum, denser to confluent laterally, disc poorly sculpted; abdomen with horseshoe sculpture, median part almost smooth, denser laterally near the margin; posterior coxae reborded laterally, setigerous sculpture made of transversal to backward-curved striae; mesosternal apophysis glabrous, transverse with minute points, strongly compressed between mesocoxae but not protruding; male abdomen concave with visible groove on sternites 2–5.

*Legs.* From dark brown to chestnut brown, with whitish pilosity; protibiae bidentate, meso- and metatibiae with median carina; profemora strigillate, mesofemora with crescent punctures or small striae, long setigerous stria along internal margin; metafemora slightly dilated with crescent punctures or small striae; all tarsal segments longer than first, metatarsi spiny, claws normal.

*Aedeagus.* Parameres fused and short, with two carinated lateral spines at apex, apical centre with short protrusion.

#### Remarks.

Only the MNHN type specimens are known. No recent material was found in the collections examined. Unfortunately, Lansberge (1882) did not specify the number of specimens used for his description. The specimen length indicated in his work does not match the measurements reported above. This difference cannot be explained at this stage, but it is possible that Lansberge (1882) may have only provided a coarse estimate, without accurate measurement. The female is larger than the male; its abdomen does not exhibit a deep groove but there are occasional sligth depressions on sternites III and IV, otherwise it varies from flat to slightly convex. The main difference between the two sexes lies in the metatibial spurs, which are strongly enlarged in the female (especially the upper one, spatuliform when thin), but acute and curved at the apex in the male. Nothing is known about the biology of this species, but the adult is probably a flower visitor.

### Atrichelaphinis (Heterelaphinis) sexualis

Taxon classificationAnimaliaColeopteraScarabaeidae

(Schein, 1956)

Figure 6

Leptothyrea sexualis Schein, 1956: 196; Marais and Holm 1992: 42; Krajcik 1998: 52.

#### Type specimen.

Holotype in NMKE: "Somaliland, Wardere, V.19 (THE Jackson)".

#### Translation of original description

**(n = unknown).** After Schein (1956: 196–197). Size: length 10–11 mm; width 5–6 mm. Shiny and black species.

*Head.* Clypeus longer than wide; lateral and anterior margins reborded and upturned, anterior margin flat and bilobed; deeply punctured; antennal club slightly longer than basal antennomeres, antennae orange/red.

*Pronotum.* Black or red, with white stripe along the lateral margin and two deep and round white maculae at base in male, red and without white maculae in female; almost as long as wide, posterior margin almost straight in front of scutellum, only slightly concave; posterior angles very blunt; lateral margin parallel in distal part, then strongly convergent in front.

*Scutellum.* Longer than wide, with lateral margins slightly concave, apex not acute.

*Elytra.* Black, with white macula at umbone (reaching the suture); 4–6 irregular stripes of broken white maculae and two white longitudinal stripes on disc, parallel to suture, made of irregular and interrupted spots in male; female without white maculae or only reduced marks in place of male stripes; white macula at sutural apex most often present; lateral margins subparallel, narrowing slightly towards apex; lateral costa forked and raised near the shoulder, reaching the humeral callus; sutural costa raised; third costa between sutural and lateral equally raised, joining the lateral costa near apical callus; suture and costae smooth, intervals exhibiting two thin geminate striae usually dissipating near lateral declivity.

*Pygidium.* Orange/red, covered by annular and ovoid sculpture; with two elongate and interrupted white maculae (separated in 4 parts) in male, absent in female.

*Underside.* Black, with last and penultimate segments orange/red in female; white maculae on epimeres, lateral parts of sternum and laterally on abdominal sternites 2–5 in male; female immaculate; fore coxae and sides of sternum with whitish pilosity; metasternal apophysis constricted between metacoxae, anterior part flat in shape of hammer; metasternum smooth at middle towards median sulcus, sides striated; abdominal segments widely punctated, with thin setae on sides; male without mid abdominal depression.

*Legs.* Protibiae widened towards apex, second tooth rounded in male, acute in female; metatibial spurs uneven and acute (longer one slightly curved) in male, enlarged with blunt apex in female; tarsi slender, metatarsus as long as as metatibia in male, shorter in female, first tarsal segment not spiny in either sexes.

*Aedeagus.* Apex of parameres round with two very small and short median protrusions, without space between them.

#### Remarks.

Described from Somaliland with no specification on number of type specimens. The description is based on the work of Schein (1956: 196–197) but no further information could be obtained on the types studied by Schein. Also, no newly collected specimens were obtained during this study. The species seems to be restricted to the Ogaden region along the south-eastern Ethiopian border with Somalia. The biogeographic characteristics of the area suggest that the species may be present in both countries. Like the other species, it is probably a flower visitor.

**Figure 6.**
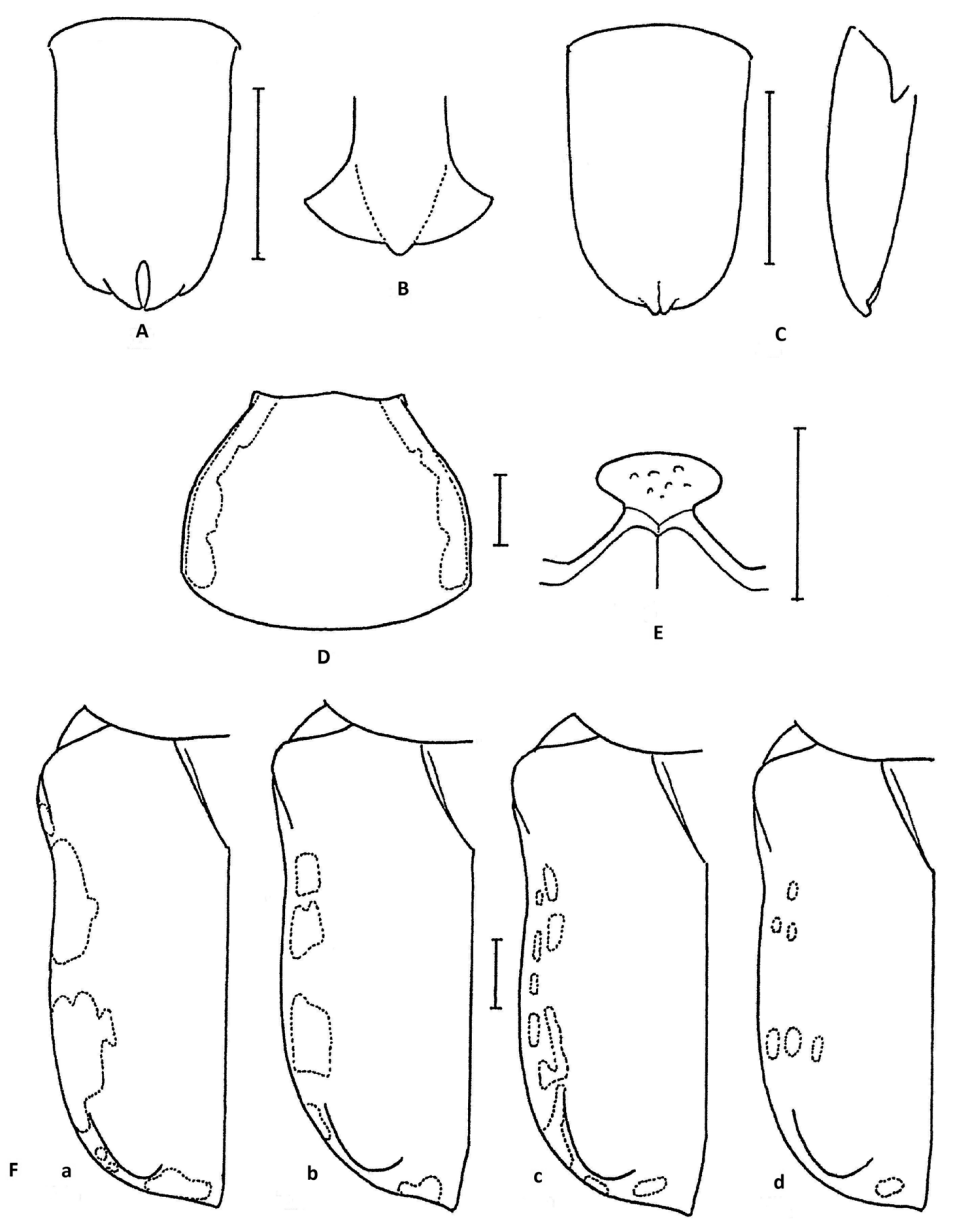
Atrichelaphinis (Heterelaphinis) sexualis: **A** parameres. Atrichelaphinis (Heterelaphinis) quadripunctata: **B** apex of the parameres of the the lectotype. Atrichelaphinis (Heterelaphinis) nigra: **C** parameres **D** male pronotum **E** mesosternal lobe **F** left elytron (**a** male; **b–d** female). Scale bar = 1 mm (From Antoine 2002: 187; permission obtained: 13 Feb 2014).

### Atrichelaphinis (Heterelaphinis) nigra

Taxon classificationAnimaliaColeopteraScarabaeidae

Antoine, 2002: 185

Figures 6 and 7

#### Type specimens.

Holotype male in MNHN: "Somalie, Berbera Check, ex. Coll. Argod 1931". Two female paratypes in MNHN with the same label.

#### Redescription

**(n = 3).** Size: length 8.8–10.3 mm; width 5.2–6.0 mm.

*Body.* Appearance stocky, black to dark-brown, from dull to slightly shiny, with white tomentose spots; lateral and irregular band on pronotal margin in male, narrower in female, occasionally reduced to line on lateral angle; three main spots on lateral margins of elytra in male, reduced and fragmented in female.

*Head.* Longer than wide, rectangular, with slightly sinuate anterior margin, slightly upturned and markedly thickened; disc convex; sculpture of large and deep punctures forming laterally some striae; lateral margin almost carinate at base, curving downwards and depressed at middle and reborded in apical part, as anterior margin; vertex and frons without protuberance, with same sculpture as clypeus; antennae dark-brown with club as long as flagellum in male, shorter in female.

*Pronotum.* Slightly transverse, larger at posterior angles; sculpture of transverse punctures with circular distribution centered at middle of posterior margin, middle unsculpted longitudinal line on disc, posterior margin in front of scutellum also unsculped; anterior margin slightly wider than head, slightly tectiform, lateral margins reborded with very smooth lateral angles at middle; posterior margin convex, straight to convex in front of scutellum.

*Scutellum.* Black to dark-brown, longer than wide, apex acute, smooth, with few punctures only on lateral angles and along lateral parts of basal third; grooved laterally.

*Elytra.* Dull, except costae and callus which are slightly shiny; strongly sculpted with two different punctures, fine on costae and horseshoe with central point (semi-annular) on remaining surface; sculpture of first two interstriae becoming confluent in apical half; costae strongly elevated, discal one almost complete to apical callus and strongly developed; apex angular but not produced; lateral margin reborded on basal half.

*Pygidium.* Transverse with horseshoe setigerous sculpture, setae thin and separate; medial line strongly convex, wide and smooth area just before apex reborded and depressed, depression exhibiting striae; two small depressions near anterior angles and one spot of white tomentum on each side.

*Underside.* With scattered lunulate setigerous sculpture, setae longer on sternum than on abdomen; wide crescent punctures on metasternum, disc poorly sculpted (few fine punctures), denser to confluent laterally; abdomen with horseshoe sculpture regularly distributed; posterior coxae reborded laterally, latero-posterior angles well marked, setigerous sculpture of transversal to backward-curved striae; mesosternal apophysis transverse with few setigerous punctures, compressed between the mesocoxae and not protruding; male abdomen concave with visible groove on the sternites 3–5; two small lateral spots on sternite 6 in male, absent in female.

*Legs.* Exhibiting whitish double setae, one long and simple, second scale-type; protibiae bidentate, meso- and metatibiae with carina in apical third; profemora strigillate, mesofemora with crescent punctures to small striae, long setigerous stria along internal margin; metafemora slightly dilated, with crescent punctures to small striae; first tarsal segment shorter than others, metatarsi not spiny; claws normal.

*Aedeagus.* Simple, with sides converging in front; apex rounded and slightly protruding at center, very short longitudinal incision just in front of protrusion.

#### Remarks.

This species is only known from the type series (male holotype and two female paratypes) and is apparently restricted to Somalia. Females exhibit a convex abdomen and enlarged to spatuliform metatibial spurs, while male spurs are slender and acute.

**Figure 7.**
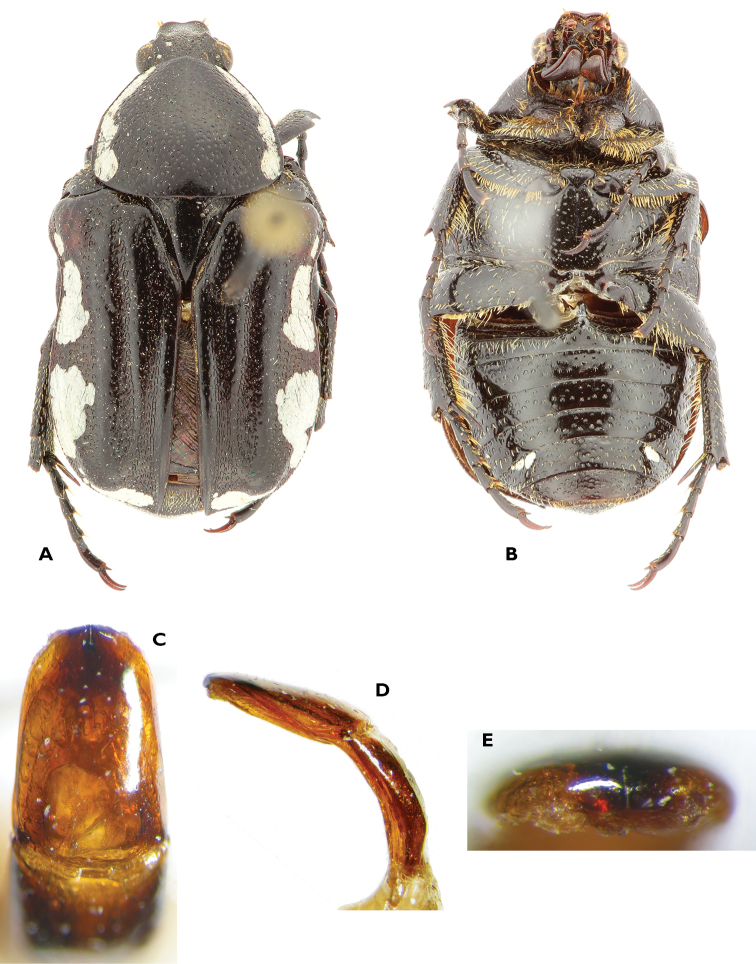
Atrichelaphinis (Heterelaphinis) nigra Antoine, 2002. Holotype male, Somalia (MNHN). **A** Dorsal view **B** ventral view **C** parameres in dorsal view **D** parameres in lateral view **E** apex of the parameres.

### Atrichelaphinis (Eugeaphinis) subgen. n.

Taxon classificationAnimaliaColeopteraScarabaeidae

#### Type species.

*Atrichelaphinis deplanata* Moser, 1907

#### Subgeneric characters.

Clypeus transverse, more or less upturned (this represents a very strong sexual dimorphic character in some species), with anterior angles at the level of the antennal insertion; anterior pronotal border from slightly tectiform (minimally in male) to tuberculate; protibiae bidentate, with denticles normally separated; metatibial apical spurs enlarged or not in female; parameres of aedeagus fused, with apex sometimes sinuate or projecting laterally into hook-like expansion, but without frontal protrusion at middle and never reployed on ventral side, only curved downwards at apex.

The type of *Atrichelaphinis deplanata* was labelled by Antoine (1988) as "*Pseudanelaphinis deplanata*". Specimens of the same species and of *Anelaphinis kwangensis* Burgeon, 1931 were also labelled as "*Pseudanelaphinis*". However, no publication relating to this genus (*in litteris*) could be traced during this study. Eight species and one subspecies are currently included.

### Atrichelaphinis (Eugeaphinis) deplanata deplanata

Taxon classificationAnimaliaColeopteraScarabaeidae

(Moser, 1907)

Figures 8 and 9

Atrichelaphinis deplanata Moser, 1907: 316–317; Schenkling 1921: 304; Girard 1993: 165; Marais and Holm 1992: 11; Joly 1993: 9.
Atrichelaphinis deplanata (Moser, 1908) Touroult & Le Gall, 2001: 34.
Atrichelaphinis deplanate (Moser) Antoine, 1988: 48.
Atrichelaphinis kwangensis (Burgeon) Marais & Holm, 1992: 11.
Anelaphinis deplanata (Moser) Antoine, 1991: 2; Antoine 2002: 186.
Anelaphinis kwangensis Burgeon, 1931: 221–222; Burgeon 1932: 95; Burgeon 1935: 470; Basilewsky 1955: 114; Antoine 1988: 48; Antoine 1991: 2.

#### Type specimens.

*Atrichelaphinis deplanata*, holotype in MNHU: "Dahomey"; *Atrichelaphinis kwangensis*, holotype in MRAC: "Musée du Congo, Kwango V-1927, (D? Zoballo), Don R. Mayné".

#### Redescription

**(n > 30).** Size: length ♂, 9.6–13.3 mm; ♀, 9.8–12.4 mm; width ♂, 5.6–6.9 mm; ♀, 6.2–7.0 mm.

*Body.* Dorsally velutinous, background colour from light-yellow to light-brown, with many black/dark brown markings and small white maculae; scale pilosity mainly on ventral suface, more extensive in male than in female, particularly dense on antero-lateral borders of pronotum, on mesepimerons and legs.

*Head.* Clypeus transverse, almost bilobed in front, anterior margin reborded, anterior angles rounded, lateral angle visible from above, large and dense simple punctures on disc, laterally wrinkled; small white maculae scattered throughout dorsal surface, scale pilosity laterally behind eyes; antennae concolor, with clubs slightly longer in male than in female.

*Pronotum.* Exhibiting strong development of black markings, reducing the background colour to margins in some specimens; octagonal, anterior margin from straight to slightly tectiform, disc bulbous in front and without punctures; lateral margin almost completely reborded, with posterior half straight from subparallel to convergent, lateral angles rounded but visible, posterior angles rounded; posterior margin straight to weakly convex laterally, medial part strongly emarginate in front of scutellum.

*Scutellum.* With apex from weakly rounded to acute, lateral margins from straight to weakly concave and with lateral grooves.

*Elytra.* Usually showing transverse area lighter than base and apical parts, which exhibit more black marks; tricostate, with the second costa raised only in basal half; three pairs of geminate striae, sculpture of horseshoe-like punctures diverging at basal part of each stria, becoming confluent and geminate on upper half; apico-sutural angle acute, longer in male than in female.

*Pygidium.* Light brown with black markings, with horseshoe punctures and dense scale pilosity; exhibiting many depressed areas.

*Underside.* Brown and black with white maculae; scale pilosity dense on lateral parts of sternum which are striated; abdominal pilosity thinner and reduced to lateral sides where punctuation consists of few horseshoe setigerous puncture; middle of sternum and abdomen without punctuation, only longitudinal line visible on metasternum, abdomen weakly concave in male, convex in female; mesosternal apophysis transverse, compressed and not protruding between mesocoxae, metasternal declivity with scale pilosity.

*Legs.* Light brown, with scale pilosity, metafemora widened, metatibia short, thickened at apex, tarsi unmodified and normal; latero-posterior angle of metacoxae rounded; metatibial spurs thin and acute in male, slightly thickened and acute in female.

*Aedeagus.* Parameres narrowing gently towards apex, more abruptly close to apex; apex truncate and curved downwards, apical curved part from bilobed to incised (in frontal view).

#### Remarks.

**Figure 8.**
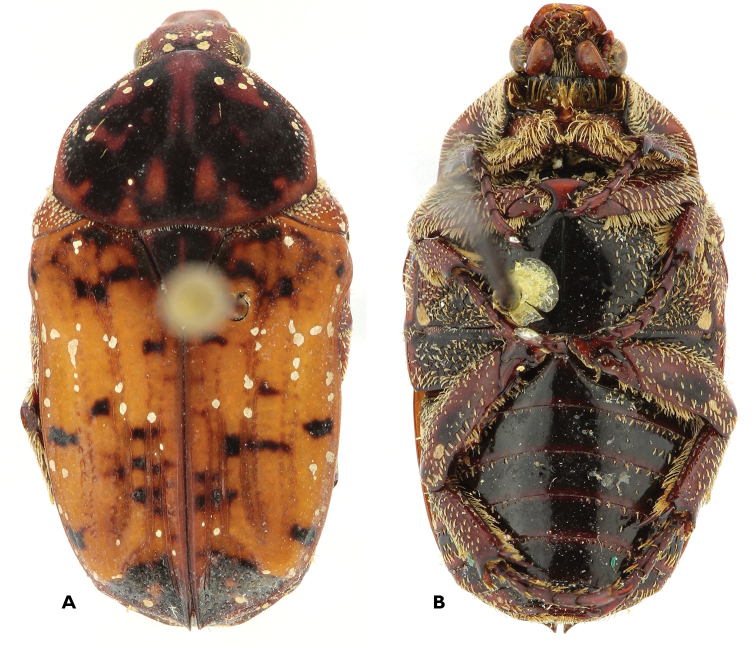
Atrichelaphinis (Eugeaphinis) deplanata deplanata (Moser, 1907), holotype (MNHU). **A** Dorsal view **B** ventral view.

Through courtesy of the MNHU and the MRAC, an opportunity was provided to study both types of *Atrichelaphinis deplanata* (Figure 7) and *Atrichelaphinis kwangensis* (Figure 8). As already indicated by Antoine (1988: 48), the synonymy between these two taxa can now be conclusively confirmed. Many specimens from several countries were analysed, including Cameroon, Guinea, Ivory Coast, Togo, Congo-Brazzaville, Congo-Kinshasa, Central African Republic and Kenya. The species seems to be a flower visitor.

**Figure 9.**
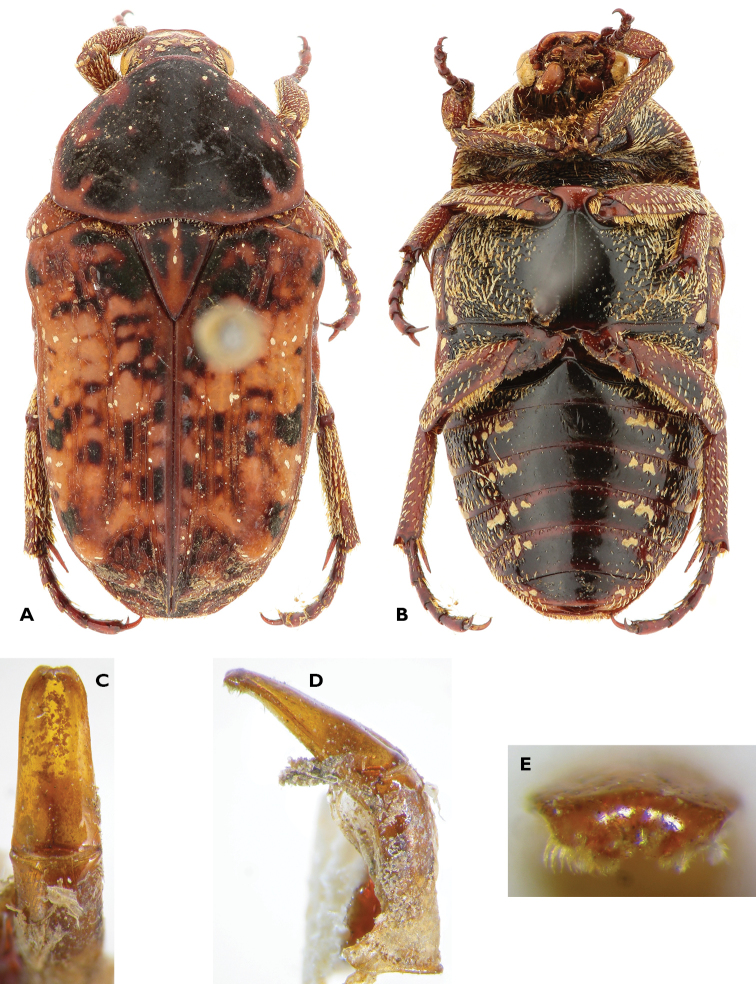
*Anelaphinis kwangensis* Burgeon, 1931, holotype (MRAC). **A** Dorsal view **B** ventral view **C** parameres in dorsal view **D** parameres in lateral view **E** apex of parameres.

### Atrichelaphinis (Eugeaphinis) deplanata minettii subsp. n.

Taxon classificationAnimaliaColeopteraScarabaeidae

Figure 10

#### Type specimens.

Holotype male, **Zambia:** Central Province, Mfwanta, S13°07'247", E30°19'345", 1429 m, R. Minetti leg., XI-2010 (MNHN ). Paratypes, **Angola:** Huila Prov., 2 km S Negola, S14°08'53", E14°28'16", à vue S. Rojkoff rec., 9-XII-2012, 1♀ (PCSR). **Congo-Brazzaville:** Pool, Mabaya, Bruno Le Rü leg, III-1989, 1♂ (PCDC). **Congo-Kinshasa:** Katanga, Manika, Ch. Seydel leg, C 19101, X-1931, 1♀ (MRAC); Kafakumba, F.G. Overlaet, IV-1932, 1♂ (MRAC); Lualaba, Zilo, Dr. V. Allard leg., XI-1974, 1♂ (MNHN Coll. Ruter); Lulua, Kapanga, F.G. Overlaet, IX-1933, 1♀ (MRAC); Katanga, exploration du PNU, riv. Kapelo, Miss. Hasson & Bouyer, Projet ICCN-NA-SEA, PNU082A, 10/16-XI-2002, 1♂ (CPTB); Katanga, exploration du PNU, env. Lusinga, Miss. Hasson & Bouyer, Projet ICCN-NA-SEA, PNU063, 25-X/5-XI-2002, 1♀ (CPTB). **Malawi:** West, Dzelanyama Fst., 4200 ft, 25-II-1985, 1♂ (PCTG); Mzuzu, Nhorongoro, S11°29’ E33°59’, 1375 m, R.J. Murphy leg, 26-XII-1996, 1♀ (PCTG); same locality, 4500 ft, R.J. Murphy leg, 30-XII-1996, 1♂ (PCTG). **Mozambique:** Sierra de Choa, D. Camiade leg, XI/XII-2012, 1♀ (PCDC). **Rwanda:** Rinkwavu, J. Roggeman leg, VI-1970, 1♂ (MRAC); Rwinkwavu, Montfort leg, IV-1976, 2♂ (IRSN, Coll. Alexis I.G. 30 374); Mayaga, J. Roggeman leg, VI-1972, 3♀ (MRAC); Kigali, Dr. V. Allard leg, II-1971, 1♂, 1♀ (MNHN, Coll. Ruter); Nyarubuye (Kibinga), Dr. V. Allard leg, 5-XII-1972, 1♂? (abdomen absent), 2♀ (MNHN, Coll. Ruter). **South Africa:** Afriq. Austr., Linokana, Dr. E. Holubi, 1894 (170–357), 1♀ (MNHN); Transvaal, ex. Coll. Oberthür, 2♂ (MNHN); Pretoria N., Van Son G., II-1936, Transvaal Mus. don, 1♀ (MRAC). **Zambia:** same data as holotype, 1♀ (PCSR); Central Province, 50 km E Serenje, S. Rojkoff & K. Werner leg, 7/8-XII-2005, 1♂ (PCSR); SE Lusaka, S15°33'662", E28°30'646", 1281 m, in fruit-baited trap, J. Touroult leg, 22-XI-2006, 1 ♀ (PCJT). **Zimbabwe:** Rhodésie du Sud, Selukwe, A. Ellenberger 1915, 1♂ (MNHN), 1♀ (PCSR).

#### Description

**(n = 31).** Size: length ♂, 9.1–11.5 mm; ♀, 10.2–11.5 mm; width ♂, 5.2–6.4 mm; ♀, 5.7–6.5 mm. This new subspecies differs from the nominal form by exhibiting the following characters: smaller size; black/brown markings more regularly disposed and reduced; background colour more reddish; anterior pronotal elevation more enhanced; lateral pronotal angles less round and hind part of lateral border slightly longer; antescutellar concavity weak; pilosity of sternum thinner, especially in male; parameres with lateral sides subparallel, apex with dull lateral angles, shape more squared.

#### Derivatio nominis.

This subspecies is named after the French collector Robert Minetti, who brought to the authors’ attention the holotype specimen from Zambia.

#### Remarks.

There was initially some reservation in erecting this new subspecies, despite the morphological differences with the other forms mentioned above. Only the study of a large series of Atrichelaphinis (Eugeaphinis) deplanata from various localities made it possible to isolate the new subspecies, considering also its broad geographic distribution. It is here given subspecies status because some of the specimens from Rwanda and Kenya actually represent a transition between the two forms, exhibitng intermediate characteristics such as coloration, shapes of pronotum and aedeagus. However, no potential intermediate forms were available from Congo-Brazzaville, where both subspecies are known to occur, but in separate parts of the country. Despite the Rwanda-Kenya transition zone, the new subspecies has a separate geographical distribution area from the nominal subspecies, which is restricted to western and central Africa. The new subspecies is distributed from central to east Africa and throughout the eastern half of southern Africa.

**Figure 10.**
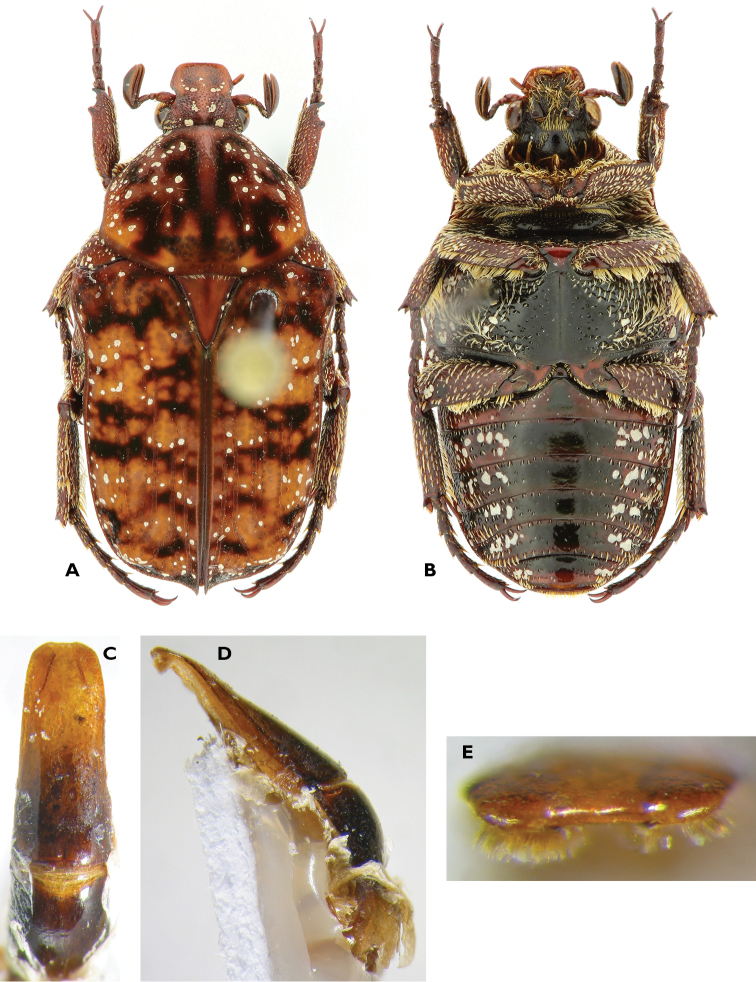
Atrichelaphinis (Eugeaphinis) deplanata minettii subsp. n., holotype (PCSR). **A** Dorsal view **B** ventral view **C** parameres in dorsal view **D** parameres in lateral view **E** apex of parameres.

### Atrichelaphinis (Eugeaphinis) rhodesiana

Taxon classificationAnimaliaColeopteraScarabaeidae

(Péringuey, 1907)

Figure 11

Niphetophora rhodesiana Péringuey, 1907: 451; Schenkling 1921: 352; Antoine 1991: 2; Holm and Marais 1992: 53.
Anelaphinis rhodesiana (Péringuey) Antoine & Holm, 1993: 102.

#### Type specimen.

Holotype male: "S. Rhodesia, Umtali " (ISAM).

#### Redescription

**(n = 42).** Size: length ♂, 10.2–12.6 mm; ♀, 10.4–12.2 mm; width ♂, 5.7–6.9 mm; ♀, 57–7.0 mm.

*Body.* Light brown mottle with dark marks from green to brown, dark color at times covering virtually entire surface; matt to shiny, white spots of tomentum scattered throughout; light pilisoty distributed on vertex, along lateral margins of pronotum, on mesepimeron, on elytra (mainly on sides and apex) and pygidium.

*Head.* Clypeus slightly transverse, anterior margin strongly upturned in male, reborded and slightly bilobed in female; disc convex; punctures scattered and superficial, striolated laterally and in front.

*Pronotum.* Transverse, lateral angles strongly rounded almost imperceptible to slightly discernible; lateral margin completely reborded; posterior margin concave in front of scutellum, laterally convex; anterior margin bluntly tuberculate at middle; punctuation sparse on disc, becoming denser and stronger laterally and in front.

*Scutellum.* With short setae and occasional round puctures at base; apex acute.

*Elytra.* With two pairs of striae between sutural costae; discolateral costae with lunulate punctures more or less complete and confluent, horseshoe sculpture also on lateral margins; apicosutural angle acute and more or less developed.

*Pygidium.* Parabolic with upturned posterior margin.

*Underside.* Shiny, generally with spots of white tomentum on abdomen and metasternum, sometimes also on metafemora; mesosternal apophysis transverse, compressed between the mesocoxae, anterior margin slightly convex; median part of metasternum and abdomen without pilosity and less sculpted.

*Legs.* Protibiae tri- to unidentate; meso and metatibae with tranverse carina under middle of external side slightly enlarged; metalegs more robust in female; second metatarsomere longer than third and fourth; with setae longer than in any other area; metatibial spurs thin and acute in male, slightly enlarged and blunt in female.

*Aedeagus.* Parameres (Figure 9D) about twice as long as wide (sometimes even longer), wider at apex than at base; laterally concave and not modified, apex convex with round angles; downturned part of apex from straight/convex to sinuate and slightly incised at middle.

#### Remarks.

A large number of specimens from Zimbabwe and South Africa was analysed for this sudy (in IRSN, MNHN, MNHU, PCRP, PCSR). The South African distribution of the species is restricted to the eastern, wetter part of the country (Holm and Marais 1992). Although no supporting records were found, the species is likely to occur also in neighbouring Mozambique and Botswana. It is normally found on a variety of flowers, fermenting fruit and sap flows.

**Figure 11.**
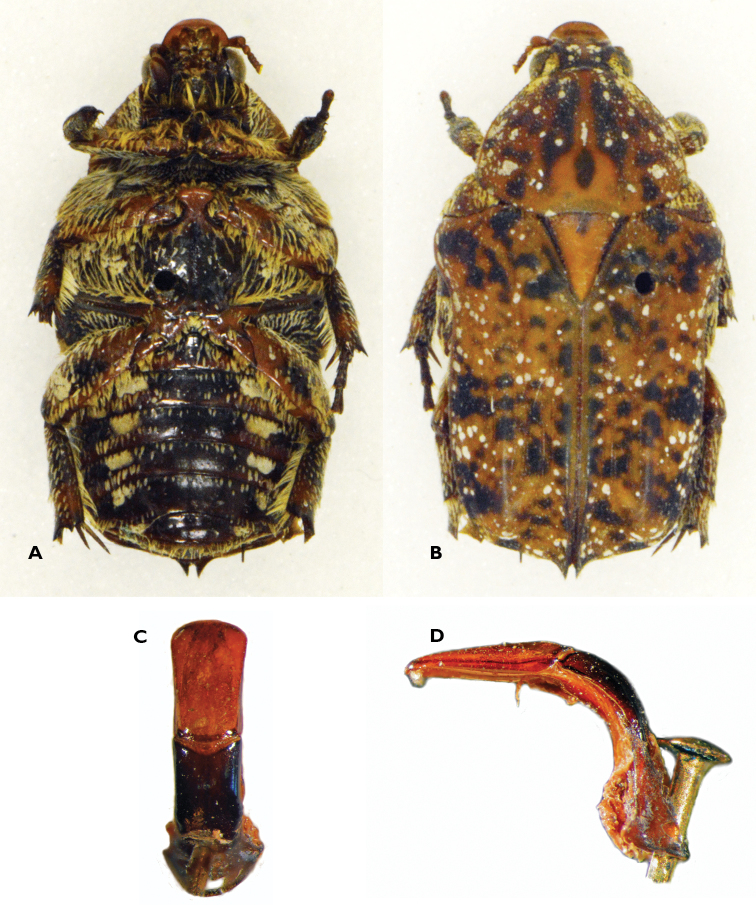
Atrichelaphinis (Eugeaphinis) rhodesiana (Péringuey, 1907), holotype (ISAM). **A** Dorsal view **B** ventral view **C** parameres in dorsal view **D** parameres in lateral view.

### Atrichelaphinis (Eugeaphinis) bomboesbergica sp. n.

Taxon classificationAnimaliaColeopteraScarabaeidae

http://zoobank.org/CE128E12-51B9-4143-8AA3-72E301FBFCB0

Figure 12

#### Type specimens.

Holotype male: **South Africa** EC, Hofmeyr, 28-XI-2011, R. Perissinotto & L. Clennell leg (ISAM). Paratypes: 1♂, same data as holotype (ISAM, PCRP); 1♂, same data as holotype, but 10-XII-2011 (PCRP); 8♂ 3♀, same data as holotype, but 24-XII-2011 (TMSA, PCSR); 2♂ 1♀, same data as holotype, but 26-XII-2011 (PCRP); 8♀, same data as holotype, but 18-XII-2010 (PCRP, PCSR).

#### Description

**(n = 25).** Size: length ♂, 9.4–11.7 mm; ♀, 10.1–12.8 mm; width ♂, 5.8–6.7 mm; ♀, 6.2 to 7.8 mm.

*Body.* Dorsal surface slightly shiny, ground colour from ochraceous to light-brown, with many black/dark brown markings and small white maculae; scale-like setae present and particularly well developed on pronotum, more extensive in male than in female.

*Head.* Anterior margin of clypeus sharply upturned, particularly in male, sligthly bilobed apically, anterior angles weakly rounded, lateral declivity visible from above; large crescent to horseshoe punctures, particularly dense on frons and vertex; scale-type setae particularly long and dense from frons to vertex; antennae with pedicel and flagellum reddish-brown, but clubs dark brown to black, club notably longer in male than in female.

*Pronotum.* With black markings not covering more than half of total area and particularly developed on anterior part of disc, on both sides of medial line; anterior margin tectiform; disc moderately tuberculate in front; with scale-like setae and round punctures diffuse but widespread thoughout surface, setae more dense and longer on lateral margins; lateral margins and angles smoothly rounded with ante-scutellar arch relatively straight.

*Scutellum.* With apex from weakly rounded to acute; lateral margins from straight to weakly concave, with shallow and narrow lateral grooves; prominent oblong medial black mark extending from base to middle of disc; exhibiting few fine punctures on apical half but no scale-like setae.

*Elytra.* Weakly tricostate, with costae barely visible in apical part; sutural costa bulging out towards middle of elytral length; striae partly geminate and with coarse horseshoe sculpture; black marking most developed around humeral and apical calluses and in mid area of lateral half; apical sutural end virtually straight in male but curving outwards in female.

*Pygidium.* Brown to reddish at centre, becoming dark brown to black towards lateral and lower margins; fine sculture and dense cover of scale-like setae throughout surface; exhibiting 2–3 pairs of depressed areas close to lateral margins.

*Underside.* Dark brown to black with white scattered maculae, particularly on metasternum and lateral margins of abdominal sternites; densely covered with long white setae, replaced in mid area of metasternum and abdominal sternites by few scattered scale-like setae; coarse and scattered horseshoe sculpture throughout, except on central areas of sternum and abdominal sternites; abdominal sternites weakly concave at middle in male, slightly convex in female; mesosternal apophysis ochraceous, small and rounded, with no projections extending between mesocoxae.

*Legs.* Tibia and femora light brown, with dark brown to black tips and joints; scattered white maculae present on both dorsal and ventral sides; tarsi dark brown to black; protibia unidentate but broadening remarkably towards apex, forming spade-like structure; numerous long setae throughout surface and scale-like setae at joints; metatibial spurs thin and acute in male, slightly enlarged and blunt in female.

*Aedeagus.* Parameres virtually straight from base to apical convergence, forming then a perfectly round apex, with slight indent at centre (dorsal view); apical margin curving downwards, but no ventral folding or projections visible in lateral view.

#### Derivatio nominis.

The species is named after the Bamboesberg mountain range of the Eastern Cape Province of South Africa, where it was discovered on its south-western slopes.

#### Remarks.

This new species represents the southernmost extension of the genus distribution range in the Afrotropical Region. *Atrichelaphinis bamboesbergica* appears to be restricted to a small area of the eastern Karoo semiarid region, where its larval stages develop exclusively in the dung middens of the antbear, *Orycteropus afer* (Pallas, 1766). Adults have a relatively short life span (2–3 weeks) and appear to be unable to feed, as none has yet been observed either on fruits, flowers or sap flows.

**Figure 12.**
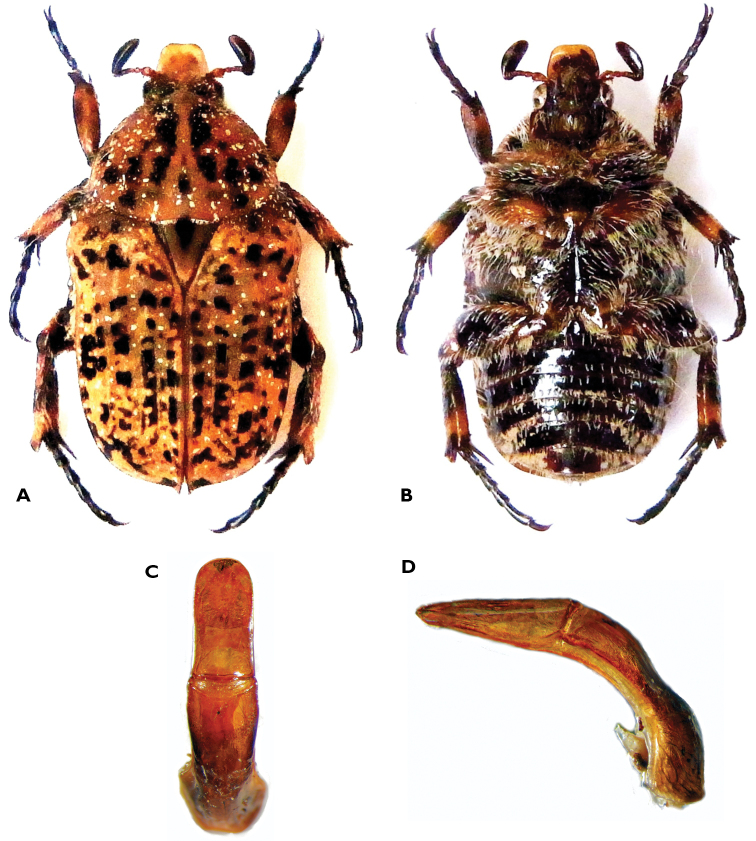
Atrichelaphinis (Eugeaphinis) bomboesbergica sp. n., holotype male (ISAM). **A** Dorsal view **B** ventral view **C** parameres in dorsal view **D** parameres in lateral view.

### Atrichelaphinis (Eugeaphinis) garnieri sp. n.

Taxon classificationAnimaliaColeopteraScarabaeidae

http://zoobank.org/8023917C-2959-465D-8EDC-A40BF9D631EC

Figures 13 and 14

#### Type specimens.

Holotype male: **Tanzania**, Mtandi Masasi reg., 19-III-2008 (IRSN). Paratypes: **Tanzania**, 2♂ 2♀, same data as HT (PCTG, PCSR, PCRP); 1♂ 1♀, same locality, but I-2006 (PCTG); 2♀, Morogoro reg., UIuguru Mts, M. Coache leg, IV-2006 (PCSR); **Zimbabwe**, 1♂, Rhod., Christon Bank, Dr. V. Allard don, 25-XI-1974 (MNHN).

#### Description

**(n = 10).** Size: length ♂, 10.7–13.6 mm; ♀, 10.7–12.9 mm; width ♂, 6.1–7.4 mm; ♀, 6.2–7.4 mm.

*Body.* Light brown mottle with dark marks from green to brown, dark color at times covering virtually entire surface; matt to shiny, white spots of tomentum scattered throughout; light pilisoty distributed on vertex, along lateral margins of pronotum, on mesepimeron, on elytra (mainly lateral margins and apex) and pygidium.

*Head.* Clypeus slightly transverse, anterior margin strongly upturned in male, reborded and slightly bilobed and upturned in female; disc convex; sculpture scattered and superficial becoming striolated laterally and in front, few setae on frons and vertex.

*Pronotum.* Transverse, lateral angles strongly rounded and from almost imperceptible to slightly discernible; lateral margin completely reborded; posterior margin concave in front of scutellum, laterally convex; anterior margin tuberculate at middle; punctuation sparse on disc, becoming denser and stronger laterally and in front; pilosity present on lateral and frontal margins.

*Scutellum.* With very thin and short pilosity, occasional round puctures at base; apex acute.

*Elytra.* With two pairs of striae between sutural costae; discolateral costae with horseshoe sculpture more or less complete and confluent, horseshoe sculpture also on lateral margins; apicosutural angle acute and moderately developed.

*Pygidium.* Parabolic, with upturned posterior margin.

*Underside.* Shiny, generally with spots of white tomentum on abdomen and metasternum, sometimes also on metafemora; mesosternal apophysis transverse, compressed between the mesocoxae and with anterior margin slightly convex; median part of metaseternum and abdomen without pilosity and poorly sculpted.

*Legs.* Protibiae from bi- to tridentate (third tooth sometimes drastically reduced); meso and metatibae with tranverse carina under middle of external side; metafemora and metatibiae strongly enlarged in both sexes; second meta-tarsomere longer than third and fourth; male metatibial spurs large and acute, especially upper one; protarsi (excluding claws) longer than protibiae (from joint to apex of apical tooth); metatarsi robust, especially in female; metatibial spurs slightly enlarged and blunt in male, strongly enlarged to spatuliform and blunt in female.

*Aedeagus.* Less than twice as long as wide; width at base larger or equal to width at apex; lateral sides of parameres parallel to convergent, with apical margins showing sinuosity and/or hook-like projections.

#### Derivatio nominis.

The species is dedicated to the renowned French collector Thierry Garnier, who continues to contribute greatly to the knowledge of African entomofauna and brought to our attention several specimens of the type series.

#### Remarks.

Atrichelaphinis (Eugeaphinis) garnieri is very similar to Atrichelaphinis (Eugeaphinis) rhodesiana, from which it can be separated by the shape of the aedeagus and some external differences. Its body is slightly larger and the dorsal black marking is also usually darker and more developed than in Atrichelaphinis (Eugeaphinis) rhodesiana (except where forms of Atrichelaphinis (Eugeaphinis) rhodesiana do not exhibit the typical colour pattern). The pilosity of Atrichelaphinis (Eugeaphinis) garnieri is thinner and longer than that of Atrichelaphinis (Eugeaphinis) rhodesiana, especially on the underside but more difficult to appreciate on the upperside due to wear. The pronotal tubercule is also more pronounced and larger in Atrichelaphinis (Eugeaphinis) garnieri than in Atrichelaphinis (Eugeaphinis) rhodesiana. The male metatibial spurs are larger and blunt in Atrichelaphinis (Eugeaphinis) garnieri, especially the upper one, while in the female they are are spatuliform. Finally, apart form exhibiting sinuosity and/or hook-like projections, the mean ratio of length to width of the aedeagus of Atrichelaphinis (Eugeaphinis) garnieri is 1.59 versus the 1.88 of Atrichelaphinis (Eugeaphinis) rhodesiana. The two species appear to be cryptic and are sympatric in Zimbabwe, which represents the northernmost geographic limit of Atrichelaphinis (Eugeaphinis) rhodesiana and the southernmost for Atrichelaphinis (Eugeaphinis) garnieri. The two females from the Uluguru Mountains show less enlarged metatibial spurs and very sligth differences in the formation of subcoxite IX. However, it is likely that these constitute simple population variations.

**Figure 13.**
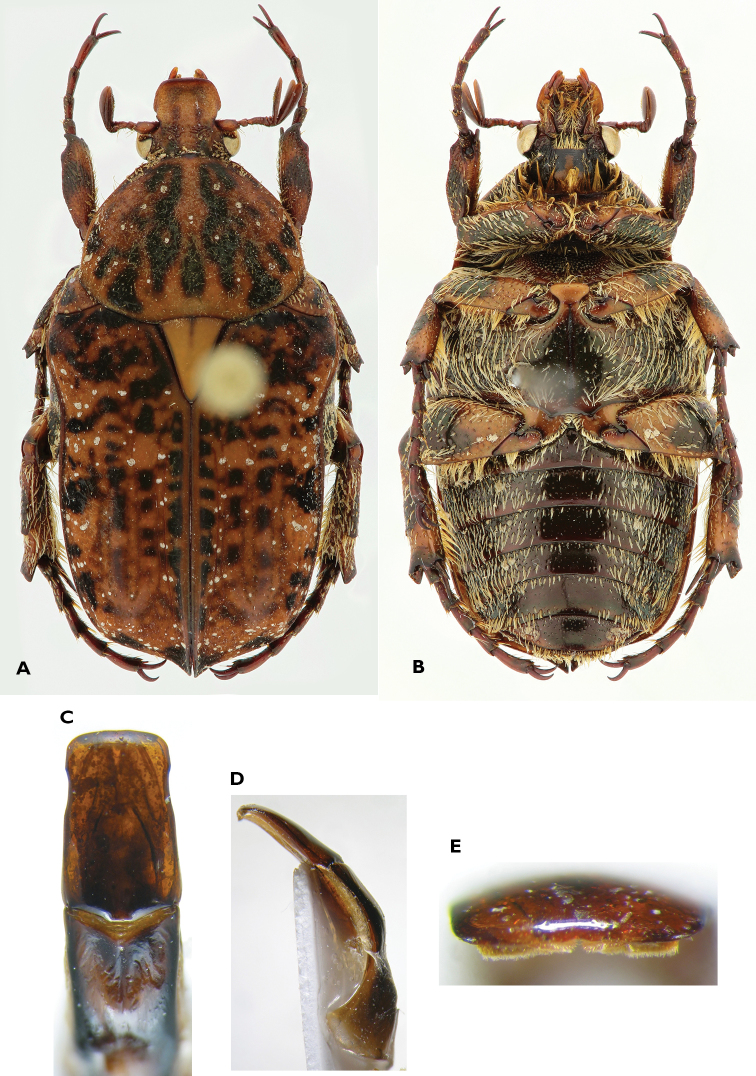
Atrichelaphinis (Eugeaphinis) garnieri sp. n., holotype male (PCSR). **A** Dorsal view **B** ventral view **C** parameres in dorsal view **D** parameres in lateral view **E** apex of parameres.

**Figure 14.**
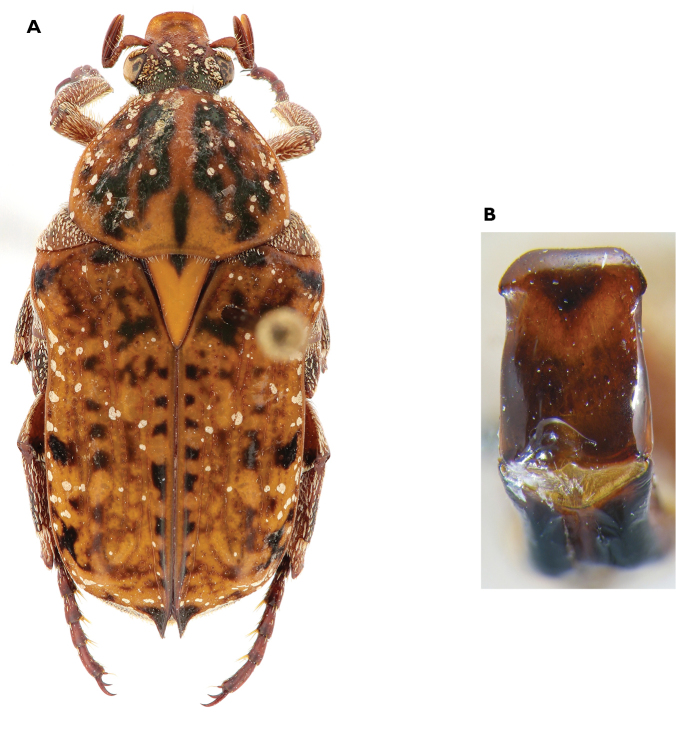
Atrichelaphinis (Eugeaphinis) garnieri sp. n., paratype male, Zimbabwe (MNHN). **A** Dorsal view **B** parameres in dorsal view.

### Atrichelaphinis (Eugeaphinis) simillima

Taxon classificationAnimaliaColeopteraScarabaeidae

(Ancey, 1883)

Figure 15

Elaphinis simillima Ancey, 1883: 94–95; Kraatz 1892: 415; Kolbe 1892a: 136; Schenkling1921: 306; Bourgoin 1930: 15; Müller 1939: 298; Antoine 1991: 2; Marais and Holm 1992: 7.
Anelaphinis simillima (Ancey) Schenkling, 1921: 306; Burgeon 1931: 219; Kolbe 1892a: 136; Müller 1939: 298; Arrow 1940: 4, 6; Marais and Holm 1992: 7.
Atrichelaphinis simillima Müller, 1939: 299.

#### Type specimen.

Holotype not traced; described from "Abyssinie" (collected by Raffray, with no date but probably ca 1881).

#### Redescription

**(n > 350).** Size: length ♂, 9.4–13.4 mm; ♀, 10.3–14.8 mm; width ♂, 5.5–7.8 mm ♀, 6.1–8.7 mm.

*Body.* Ligth brown, velutinous to shiny with dark markings never covering whole surface, always lighter areas present; mesepimeron with setigerous sculpture in both sexes.

*Head.* With metallic sheen; vertex velutinous and hairy, sometimes reaching clypeal disc; clypeus transverse, reborded and slightly bilobed in front, sometimes weakly upturned; sculpture dense and deep, simple on disc and more or less confluent in front and latetally; vertex with smooth area and tomentum, large and smooth longitudinal carina extending from vertex to clypeal disc which is convex.

*Pronotum.* Exhibiting metallic sheen and setae on lateral margins; with lateral angles usually broadly rounded, sometimes almost undistinguished; lateral margin completely reborded, posterior margin weakly concave in front of scutellum, laterally convex towards posterior angles; diffuse tomentose lines along lateral margins, sometimes very reduced, two radial lines on each side of midline usually made of three groups of spots more or less developed; sculpture marked, not dense on disc, more or less confluent laterally; dark green marks sometimes very reduced.

*Scutellum.* Longitudinal with apex from acute to blunt, without sculpture except near angles, without tomentum; with lateral grooves and sides almost straight or weakly curved inwards.

*Elytra.* With weak posthumeral emargination, reborded laterally; dark green marks sometimes very reduced or absent, but never covering whole surface; disc without tomentum; sculpture variable in size and intensity, usually vertical series of horseshoe punctures, sometimes confluent; short setae on lateral declivity and apex; apex acute but not protruding backwards.

*Pygidium.* With short setae occasionally throughout surface.

*Underside.* Shiny, with metallic sheen; with dense pilosity; mesosternal apophysis transverse, finely punctate and glabrous, not or slightly protruding in front of mesocoxae, not clearly oriented in lateral view; metasternum and abdomen sculpted laterally (horseshoe to striolate punctures) and showing white tomentum usually more developed in male; abdomen concave in male, flat or slightly convex in female.

*Legs.* Meso- and metatibiae with carina on external side just under middle; metatarsomeres shorter and more robust in female; protibiae enlarged, metatibiae slightly broader and hind spurs enlarged in female; metatibial spurs thin and acute in male, slightly enlarged and less acute but not blunt in female.

*Aedeagus.* Parameres about twice as long as wide, side from parallel to slightly convergent in front, apex truncate with lateral angles rounded, sometimes weakly bulbous laterally, emarginated at middle of downturning apical part.

#### Remarks.

All specimens examined originated from Ethiopia. Some are labeled "Shoa-Somali" but without precise locality and were collected during the expedition of V. Erlanger. They were probably also collected within the current borders of Ethiopia. The type of Ancey (1883) could not be traced. Three specimens from the Oberthür Collection, collected in "Abyssinie" by Raffray and identified as *Anelaphinis simillima* by Antoine (1992), were found at the MNHN. Two of them are "ex-Museo D. Sharp 1890" and "ex-Museo Van Lansberge", respectively. They are both bigger than the size given by Ancey in his description. The third one, labelled "Abyssinie Raffray Voy. 1881" match the description and the sizes indicated by Ancey. It is not known if this specimen is the holotype or a cotype, but a red label indicating this possibility has now been attached to it by Rojkoff (2014). Because both collections of Ancey and Raffray were scattered through different collections, it is virtually impossible to establish the precise status of this specimen. The identification of Atrichelaphinis (Eugeaphinis) simillima was based on specimens (4♂, 4♀) held in the IRSN and carrying the following labels "Comp. par Bourgoin au type" / "Harrar Abyssinie / Juin Juill. 1911 / G. Kristensen", and identified as "Elaphinis simillima Ancey / 1913 Det. A. Bourgoin". Horn et al. (1990: 18) reported that Ancey’s Cetoniinae were in the JM Bédoc/Paris Collection, but they have not been traced since. Some specimens kept in the MNHN collections are labelled "Abyssinie/Raffray", but they cannot be regarded as type material. In the same publication where Atrichelaphinis (Eugeaphinis) simillima was described, Ancey (1883) also included *Gnathocera costata* Ancey, 1883, the type material of which is housed in the MNHU, according to Marais and Holm (1992: 33).

**Figure 15.**
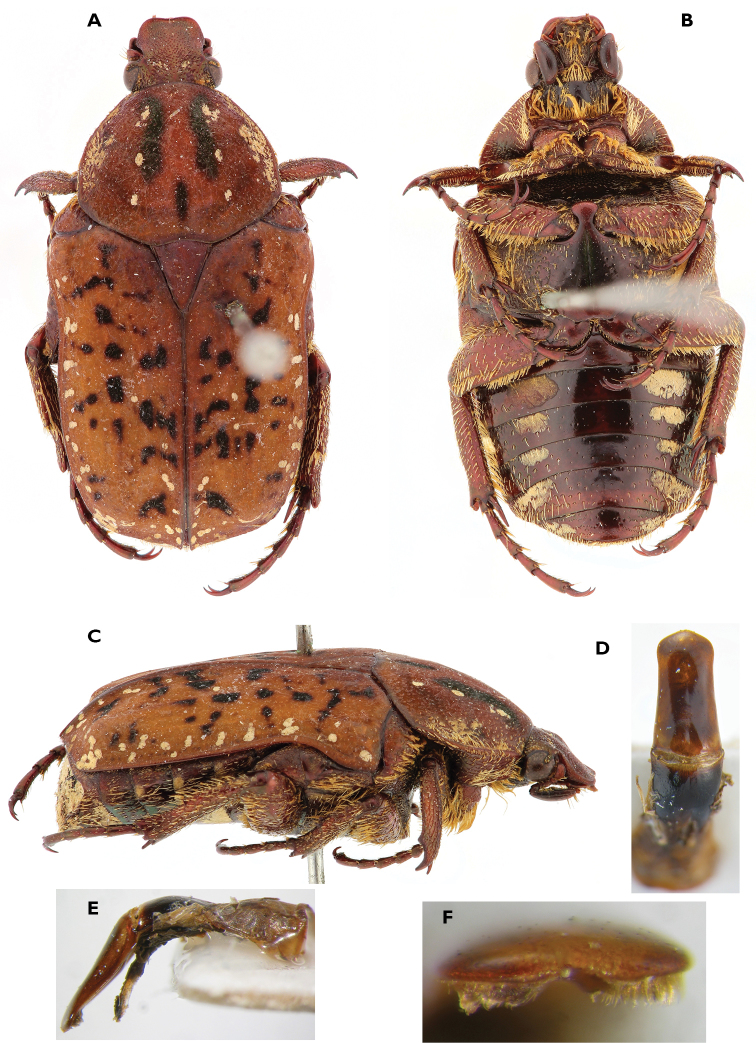
Atrichelaphinis (Eugeaphinis) simillima (Ancey, 1883), "compared to type" by Bourgoin (IRSN). **A** Dorsal view **B** ventral view **C** lateral view **D** parameres in dorsal view **E** parameres in lateral view **F** apex of parameres.

### Atrichelaphinis (Eugeaphinis) sternalis

Taxon classificationAnimaliaColeopteraScarabaeidae

(Moser, 1914)

Figure 16

Anelaphinis sternalis Moser, 1914: 606–607; Schenkling 1921: 306; Antoine 1991: 2; Marais and Holm 1992: 7.

#### Type specimen.

Holotype male: "Abessinien" (MNHU).

#### Redescription

**(n = 24).** Size: length ♂, 12.1–13.7 mm; ♀, 12.9–13.5 mm; width ♂, 7.1–8.2 mm; ♀, 7.5–7.8 mm.

*Body.* Gound color from brown orange to brown red, with green marks more or less developed, at times covering whole dorsal surface with exception of few areas of ground color; velutinous, with metallic reflections as in Atrichelaphinis (Eugeaphinis) simillima; tomentum and pilosity well developed and with almost same distribution as in Atrichelaphinis (Eugeaphinis) simillima; mesepimeron mainly glabrous and without sculpture in male (sometimes with tomentum), with setigerous sculpture in female.

*Head.* Vertex velvety sometimes reaching clypeal disc; clypeus transverse, reborded and slightly bilobed in front; sculpture dense and strong, simple on disc and more or less confluent in front and laterally; vertex with smooth area and tomentum; large and very smooth vertical carina extending from vertex to clypeal disc, which is convex.

*Pronotum.* Transverse; lateral margins with very rounded lateral angles and regularly curved from posterior to anterior angles, reborded except in front of posterior angles; posterior margin strongly concave in front of scutellum, then bisinuate on each side; sculpture very light, sometimes undiscernible, scattered on disc but slightly denser near the anterior angles, punctuation stronger in female; tomentose line along outer margins and two radial lines of three spots each at side of midline, sometimes extra spots between these and outer ones.

*Scutellum.* Longitudinal, acute to blunt, usually smooth but with few punctures in some specimens; grooved laterally, sides almost straight.

*Elytra.* With weak posthumeral emargination, reborded laterally; disc without tomentum and with sculpture consisting of simple to crescent small punctures forming simple striae and interstriae; dense horseshoe sculpture laterally, near humeral callus and apically; sutural apex acute, slightly protruding backwards in male but not in female.

*Pygidium.* With large tomentose spots and bands.

*Underside.* Shiny, with large tomentose areas on prosternum, procoxae, mesepimeron, metepimeron, metepisternum, sides of metasternum and abdomen; mesosternal apophysis finely punctate, not transverse, almost as wide as long, protruding in front of mesocoxae and orientated downwards; abdomen slightly concave in male and convex in female.

*Legs.* Shiny; meso- and metatibiae with carina on external side just below middle; female with protibiae and metatibial spurs enlarged, metatibiae stronger, metatarsomeres shorter and more robust than in male; metatibial spurs very thin and acute in male, very slightly enlarged and less acute but not blunt in female.

*Aedeagus.* Length of parameres less than twice their width, sides converging in front, apex rounded, not truncate and not bulbous laterally, incised in the mid downturning part of apex.

#### Remarks.

This species is currently only known from Ethiopia. It is very close to Atrichelaphinis (Eugeaphinis) simillima from which it can be separated through the sculpture of the dorsal side, the shape of the mesosternal apophysis and, to a lesser extent, the aedeagus. The Atrichelaphinis (Eugeaphinis) species from Ethiopia are sometimes difficult to identify. For example, the Alexis Collection (IRSN) holds specimens from Lake Tana that exhibit a color pattern typical of Atrichelaphinis (Eugeaphinis) sternalis; however upon close scrutiny they were found by the authors to resemble most closely Atrichelaphinis (Eugeaphinis) simillima. However, the general body shape, the laterally bulbous apex of the parameres, the slightly more upturned anterior margin of the clypeus and the very weakly protruding mesosternal apophysis in front of the mesocaxae without downturning could cast some doubt over this identification. All the other characters are similar to those found in Atrichelaphinis (Eugeaphinis) simillima. It is also possible that these specimens could represent either a new species, subspecies or just an hybrid between the two species. Another possibility is that of marked intraspecific variation. It may be necessary to study extensive series of specimens from more localities in order to resolve this issue conclusively.

**Figure 16.**
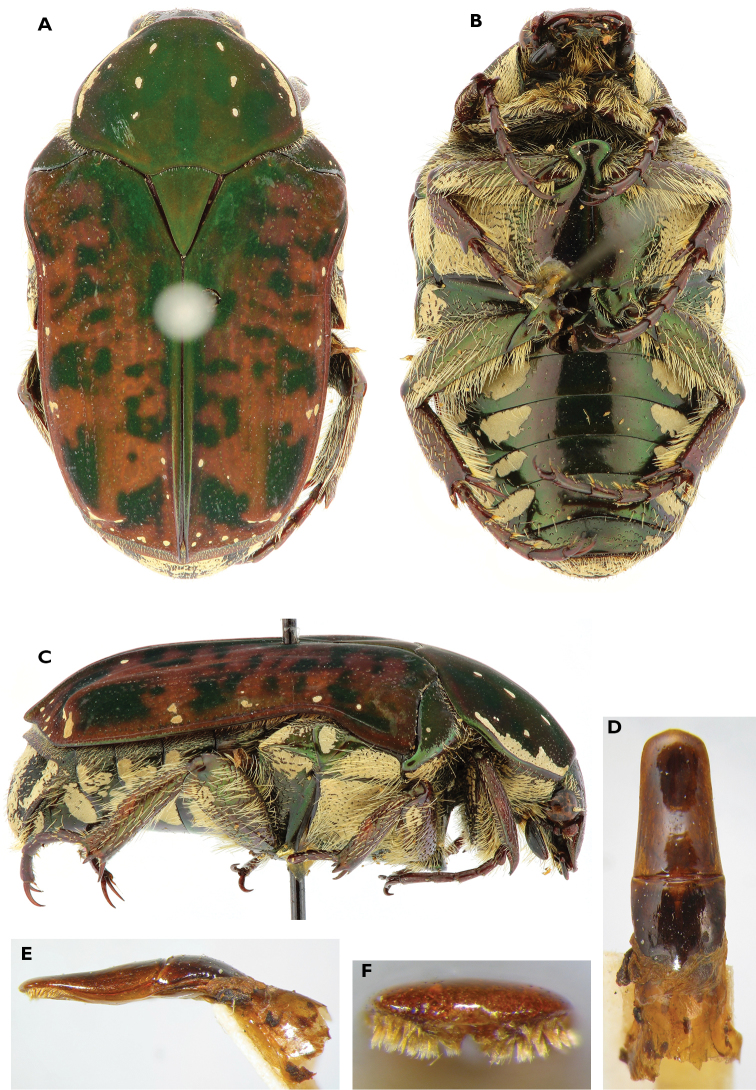
Atrichelaphinis (Eugeaphinis) sternalis (Moser, 1914), holotype (MNHU). **A** Dorsal view **B** ventral view **C** lateral view **D** parameres in dorsal view **E** parameres in lateral view **F** apex of parameres.

### Atrichelaphinis (Eugeaphinis) vermiculata

Taxon classificationAnimaliaColeopteraScarabaeidae

(Fairmaire, 1894)

Figure 17

Elaphinis vermiculata Fairmaire, 1894: 653–654; Kraatz 1895a: 381; Kraatz 1895b: 384; Preiss 1902: 99; Schenkling 1921: 304; Antoine 1991: 2; Antoine 2002: 186.
Anelaphinis vermiculata (Fairmaire) Antoine, 2002: 186.
Atrichelaphinis vermiculata (Fairmaire) Kraatz, 1898: 220; Schenkling 1921: 304; Antoine 2002: 186.

#### Type specimen.

Holotype not traced.

#### Redescription

**(n = 11).** Size: length ♂, 12.1–13 mm; ♀, 10.7–13.9 mm; width ♂, 6.7–7.6 mm ♀, 6.1–7.9 mm.

*Body.* Velutinous, from light brown with dark marks to dark green with dark brown areas, white small irregular spots scattered throughout, sometimes becoming confluent on lateral declivity of elytra, pronotum and pygidium; light pilosity usually present on vertex and lateral margins of pronotum, mesepimeron, elytral apex and pygidium; mesepimeron with sculpture and pilosity limited to anterior half, posterior half smooth.

*Head.* With median vertical smooth carina extending from vertex to clypeal disc; clypeus clearly transverse, anterior margin reborded in both sexes but not strongly raised, slightly incised in the middle, more strongly punctate laterally and in front, where punctuation becomes confluent; disc exhibiting smooth areas.

*Pronotum.* Octagonal; not tuberculate on anterior margin; lateral angles well marked but rounded; lateral margins completely reborded, with posterior half parallel; posterior angles rounded; sculpture of setigerous crescents, usually sparce and poorly pronounced on disc, but stronger in front and on lateral marginss; posterior margin from straight to slightly concave in front of scutellum, lateral edges convex.

*Scutellum.* Not uniformly sculpted, laterally grooved, and with white tomentum.

*Elytra.* With lateral margins almost straight and parallel, posthumeral emargination weak; sculpture of strong and well marked horseshoe punctures sometimes confluent, especially in apical half; space between vertical lines of punctuation of same width, appearing not geminate; sutural apex blunt.

*Pygidium.* With white, small spots becoming confluent; light pilosity throughout.

*Underside.* Shiny, with small white spots on postero-lateral angles of sternites and metasternum, sometimes on the mesepimeron and metepimeron, some apical spots also on femora; pilosity long and thin, extending throughout surface except middle of metasternum and abdomen; mesosternal apophysis transverse, anterior margin straight, strongly compressed between mesocoxae and not protruding in front in lateral view; abdomen concave in male, convex in female, last sternite less sculpted at middle in male.

*Legs.* Metafemora sometimes with white spot of tomentum on underside close to joint; protibiae enlarged in female, meso- and metatibiae with transverse carina just after middle; metatibial spurs slender and more acute in male, larger and blunt in female.

*Aedeagus.* Parameres converging regularly at apex, without lateral expansions or modifications; apex with marked incision at middle of downturning margin.

#### Remarks.

Most of the specimens analysed in this study originated from Erythrea (PCDC, PCSR, MNHN, MNHU). Although the type was not traced, all specimens match Fairmaire’s (1894) original description. The species has also been reported from Ethiopia, locality confirmed through the study of one female labelled "Abyssinie, Tigray, Alitiena" (close to the Erythrean border) and one couple labelled "Abyssinien" in the MNHN collections.

**Figure 17.**
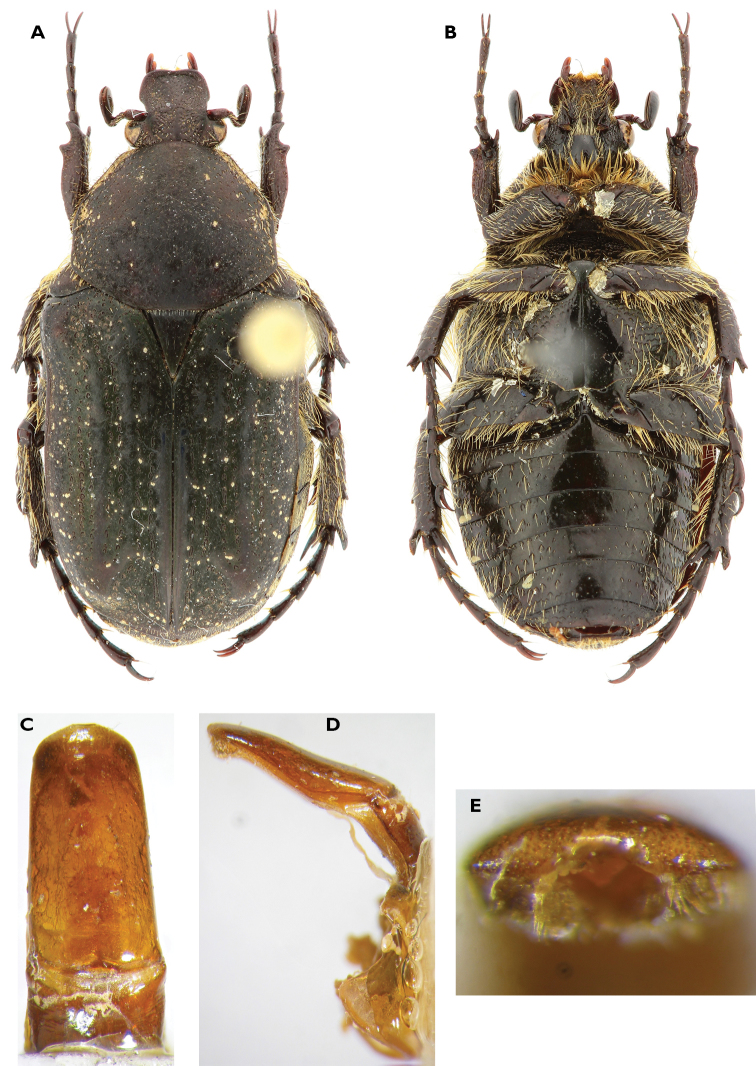
Atrichelaphinis (Eugeaphinis) vermiculata (Fairmaire, 1894), male, Erythrea (PCSR). **A** Dorsal view **B** ventral view **C** parameres in dorsal view **D** parameres in lateral view **E** apex of parameres.

### Atrichelaphinis (Eugeaphinis) bjornstadi sp. n.

Taxon classificationAnimaliaColeopteraScarabaeidae

http://zoobank.org/ACCEC445-F973-4AED-A334-875219242983

Figure 18

#### Type specimens.

Holotype male: **Tanzania**, Mbulu, Mamamisara, 2000 m, J. Kielland leg, (Bjørnstad 34728), 6-III-1981 (IRSN). Paratypes: **Tanzania**, 1♂, same data as HT (Bjørnstad 34727) (PCSR); 4♂ 1♀, Babati D., Mt. Kwaraha, 1850 m, J. Kielland leg, 30-IV-1987 (Bjørnstad 35080, PCTG; Bjørnstad 35077-35079 and 35081, PCSR, PCRP and IRSNB); 1♀, Ngorongoro Crater, 2200 m, J. Kielland leg, 14-II-1980, (Bjørnstad 35052, PCSR); 1♀ same data as above but 2300 m (Bjørnstad 41980, PCAB).

#### Description

**(n = 9).** Size: length ♂, 13.6–14.6 mm; ♀, 12–15 mm; width ♂, 7.9–8.5 mm; ♀, 7.1–8.7 mm.

*Body.* Velutinous, brown with green to dark green marks, with small white spots scattered throughout, sometimes becoming confluent on lateral declivity of elytra, pronotum and pygidium; light pilosity distributed on vertex, lateral margins of pronotum, apical part of elytra and pygidium; mesepimeron with sculpture and pilosity on whole surface.

*Head.* Clypeus slightly transverse, almost as long as wide, anterior margin reborded and slightly incised at middle; disc convex, regularly punctated on entire surface, except few small smooth areas, punctures denser and confluent laterally and in front.

*Pronotum.* Not tuberculate in front, with round and slightly detectable lateral angles; posterior half of lateral margins not parallel but convergent in front; posterior angles blunt; posterior margin strongly concave in front of scutellum, with lateral edges almost straight; sculpture of setigerous crescent punctures, almost absent on disc, denser in front and laterally.

*Scutellum.* Unsculpted, laterally grooved, with white tomentum.

*Elytra.* With lateral margins almost straight and parallel, posthumeral emargination weak; sculpture of thin and incomplete horseshoe punctures more developed laterally and at apex, sometimes confluent resulting in broken lines; lines of punctuation geminate; sutural apex acute.

*Pygidium.* With small white spots scattered throughout, becoming confluent.

*Underside.* Shiny, with white confluent tomentum laterally on anterior margin of sternites and on lateral sides of metasternum; pilosity long and thin; mesosternal apophysis transverse, anterior border slightly convex, strongly compressed between mesocoxae and not protruding in front in lateral view; abdomen concave in male, convex in female; last sternite less sculpted at middle in male.

*Legs.* Metafemora sometimes with white spots of tomentum on underside close to joint; meso- and metatibiae with transverse carina just after middle; metatibial spurs thinner and more acute in male, larger and blunt in female.

*Aedeagus.* Parameres forming slight concavity at middle of lateral margins; without projections at apex, but with marked incision at middle of downturning frontal margin.

#### Derivatio nominis.

This species is named after the Norwegian entomologist Anders Bjørnstad, who provided the type series for study.

#### Remarks.

This species is most closely related to Atrichelaphinis (Eugeaphinis) vermiculata, from which it can be separated mainly by the shape of the clypeus. It has also a distinct pronotum, with lateral margins strongly diverging in a posterior direction and the lateral angles obliterated, which also allow easy separation from Atrichelaphinis (Eugeaphinis) vermiculata. Its elytra exhibit visible but relatively shallow sculpture. The species has so far only been recorded from northern Tanzania.

**Figure 18.**
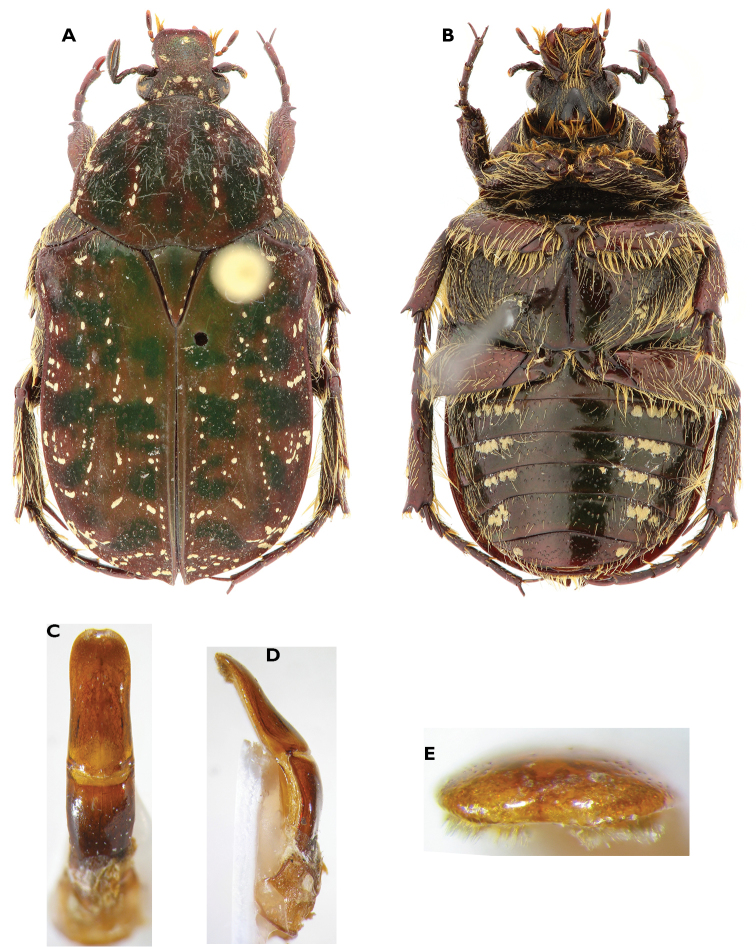
Atrichelaphinis (Eugeaphinis) bjornstadi sp. n., holotype (PCSR). **A** Dorsal view **B** ventral view **C** parameres in dorsal view **D** parameres in lateral view **E** apex of parameres.

### Updated key to the species of the genus *Atrichelaphinis* Kraatz, 1898

**Table:** 

	Protibiae tridentate, with two apical teeth close to each other	**2**
–	Protibiae bi- or tridentate, with two apical teeth normally separated	**3**
2	Pygidium with setae and small round sculpture; first two elytral striae consisting of double grooves (Figure 3)	**Atrichelaphinis (Atrichelaphinis) tigrina (Olivier, 1789)**
–	Pygidium with scattered crescent sculpture, asetose; first two elytral striae consisting of single grooves (Figure 4)	**Atrichelaphinis (Atrichelaphinis) nigropunctulata (Péringuey, 1896)**
3	Dorsum black, sometimes with red parts or with yellowish elytra; clypeus longer than wide; aedeagus with protrusion at middle of apex in dorsal view	**4**
	Dorsum never black, usually ground color light brown or green; clypeus transverse; aedeagus simple at apex, occasionally with lateral projections	**6**
4	Elytra yellowish; parameres medially and laterally protruding (Figure 5, Figure 6B)	**Atrichelaphinis (Heterelaphinis) quadripunctata (Lansberge, 1882)**
–	Elytra black to dark brown; parameres protruding only medially	**5**
5	Dorsum entirely black without red areas, mesosternal apophysis large; medial protrusion of parameres incised but without meatus (Figure 6C–F, Figure 7)	**Atrichelaphinis (Heterelaphinis) nigra Antoine, 2002**
–	Dorsum with red areas (pronotum, scutellum, pygidium and last sternites); medial protrusion of parameres more developed, deeply incised and with large meatus (Figure 6A)	**Atrichelaphinis (Heterelaphinis) sexualis (Schein, 1956)**
6	Pronotum tuberculate at middle of anterior margin	**7**
–	Pronotum not tuberculate	**9**
7	Clypeus upturned in both sexes (less in female), apical half of scutellum punctate to striolate (Figure 12)	**Atrichelaphinis (Eugeaphinis) bambooesbergica sp. n.**
	Clypeus upturned only in male, simply reborded in female, scutellum without sculpture on apical half	**8**
8	Protarsi longer than protibiae; metafemora and metatibiae strongly enlarged (Figure 13), metatibial spurs enlarged and blunt in both sex, spatuliform in female	**Atrichelaphinis (Eugeaphinis) garnieri sp. n.**
–	Protarsi shorter than protibiae; metafemora and metatibiae slightly enlarged (Figure 11), metatibial spurs thin and acute in male, slightly enlarged and blunt in female	**Atrichelaphinis (Eugeaphinis) rhodesiana (Péringuey, 1907)**
9	Mesosternal apophysis prominent between mesocoxae and projecting downwards (in lateral view), lateral margins of pronotum incompletely reborded near posterior angles (Figure 16)	**Atrichelaphinis (Eugeaphinis) sternalis (Moser, 1914)**
–	Mesosternal apophysis not prominent between mesocoxae	**10**
10	Apicosutural angle of elytra acute and projecting backward; species of small size (9–12 mm)	**11**
–	Apicosutural angle of elytra not projecting backward; species of larger size (12 to 15 mm)	**12**
11	Pronotum predominantly dark-brown, with light colour and white tomentum restricted to margins; with posterior border clearly concave in front of scutellum; elytra with two large lateral light brown areas adjacent to metacoxae, basal and apical parts dark; parameres converging towards apex, then more abruptly near apex, apex truncate in front (Figures 8, 9)	**Atrichelaphinis (Eugeaphinis) deplanata deplanata (Moser, 1907)**
–	Pronotum light in colour, dark markings reduced but white tomentose spots more widespread; posterior margin weakly concave in front of scutellum; elytra light brown with dark markings regularly distributed; parameres almost parallel towards apex, truncate in front forming blunt angles (Figure 10)	**Atrichelaphinis (Eugeaphinis) deplanata minettii subsp. n.**
12	Elytral sculpture well developed, showing series of regularly-spaced horseshoe punctures (Figure 17)	**Atrichelaphinis (Eugeaphinis) vermiculata (Lansberge, 1882)**
–	Elytral sculpture faint and incomplete, with intervals between punctures irregular	**13**
13	Posterolateral angles of metacoxae from subacute to blunt; medium size species usually with metallic sheen, elytra light brown with few, small dark marks; tomentum mainly restricted laterally on pronotum and elytra (Figure 15)	**Atrichelaphinis (Eugeaphinis) simillima (Ancey, 1883)**
–	Posterolateral angles of metacoxae widely rounded; larger species without metallic sheen on dorsum; ground colour brown with large green patches and white tomentum scattered on entire surface (Figure 18)	**Atrichelaphinis (Eugeaphinis) bjornstadi sp. n.**

### Key to the African genera of Cetoniini close to *Atrichelaphinis*, with completely or partially fused parameres.

**Table:** 

1	Parameres completely fused, except for occasional presence of small sinuosity or incision on downturning apical margin (frontal view); parameres with or without projections	**2**
–	Parameres partially fused, with apex incised or modified (dorsal view)	**5**
2	Internal sac of aedeagus with sclerites (Figure 19A)	***Heteralleucosma* Antoine, 1989**
–	Internal sac without sclerites	3
3	Protibia tridentate, with two apical teeth close to each other (Figures 3, 4)	**Atrichelaphinis (Atrichelaphinis) Kraatz, 1898**
–	Protibia bi- or tridentate, with two apical teeth widely separated; mesosternal apophysis transverse and flat	**4**
4	Aedeagus with protrusion at middle of apex (dorsal view) (Figure 5)	**Atrichelaphinis (Heterelaphinis) Antoine, 2002**
–	Aedeagus simple at apex, but often exhibiting lateral projections (Figures 6–16)	**Atrichelaphinis (Eugeaphinis) subgen. n.**
5	Internal sac of aedeagus without sclerites	**6**
–	Internal sac with sclerites	**11**
6	Parameres flat and composed of two weakly sclerotized lateral lobes, with median azygous sclerotized lamina (Figure 19B)	***Niphetophora* Kraatz, 1883**
–	Parameres not as above	**7**
7	Parameres flat	**8**
–	Parameres visibly convex in lateral view	**9**
8	Parameres with small incised protrusion at middle of apex (Figure 5)	**Atrichelaphinis (Heterelaphinis) Antoine, 2002**
–	Parameres with apical incision exhibiting two lateral, slightly sclerotized triangular parts; anterior border of clypeus separated from disc by deep groove (Figure 19C)	***Paranelaphinis* Antoine, 1988**
9	Apex of parameres with expansion visible in lateral view (Figure 19D)	***Molynoptera* Kraatz, 1897**
–	Apex of parameres not expanded	**10**
10	Apical end of parameres with sharp but thin hook visible in lateral view and protruding on ventral side (Figure 19E)	***Pseudalleucosma* Antoine, 1989**
–	Apical end of parameres without modifications visible in lateral view, round with setae on ventral side (Figure 19F)	***Molynopteroides* Antoine, 1989**
11	Internal aedeagal sac with three sclerites; parameres flat, incised at middle of apex and slightly sclerified laterally at apex (Figure 19G)	***Phaneresthes* Kraatz, 1894**
–	Internal aedeagal sac with one or two sclerites	**12**
12	Sclerites composed of two bands; parameres almost flat, slightly thickened and curved in apical third from lateral view, with apex rounded and exhibiting small median incision (Figure 19H)	***Paralleucosma* Antoine, 1989**
–	Only one sclerite present	**13**
13	Sclerite consisting of thin, ovoid, longitudinal and erect band; parameres usually with cavity on upper side just before apex, apex more or less modified at extremity, setae on ventral side virtually sclerified (Figure 1)	***Anelaphinis* Kolbe, 1892**
–	Sclerite not as above	**14**
14	Parameres subparallel, sharply narrowing before apex, sclerite with complex shape (Figure 19I)	**Alleucosma (Alleucosma) Schenkling, 1921**
–	Parameres triangular, regularly narrowing from base to upturned apex; sclerite small, oval or flat but not with complex shape (Figure 19J)	**Alleucosma (Eoalleucosma) Antoine, 1989**

**Figure 19.**
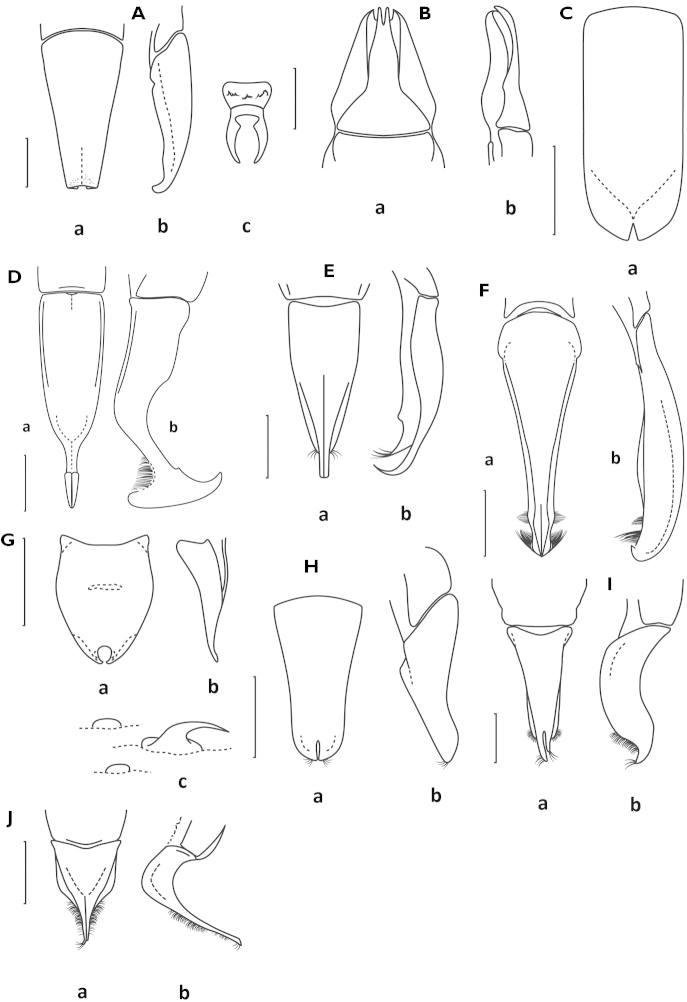
Schematic outlines of parameres (**a** frontal; **b** lateral; **c** chitinous appendage). **A** *Heteralleucosma insignis* Antoine, 1989 **B** *Niphetophora hildebrandti hildebrandti* (Harold, 1878) **C** *Paranelaphinis signata* Antoine, 1988 **D** *Molynoptera multiguttata* Kraatz, 1897 **E** *Pseudalleucosma machatschkei* (Ruter, 1960) **F** *Molynopteroides guttiventris* (Moser, 1914) **G** *Phaneresthes flavovariegata* Kraatz, 1894 **H** *Paralleucosma glycyphanoides glycyphanoides* (Moser, 1908) **I** Alleucosma (Alleucosma) viridula (Kraatz, 1880) **J** Alleucosma (Eoalleucosma) duvivieri (van der Poll, 1890) (Figure **C** after Antoine 1988; Figures **G** after Antoine 1989). Scale bar = 1 mm.

## Supplementary Material

XML Treatment for Atrichelaphinis s. l.

XML Treatment for Atrichelaphinis (Atrichelaphinis)

XML Treatment for Atrichelaphinis (Atrichelaphinis) tigrina

XML Treatment for Atrichelaphinis (Atrichelaphinis) nigropunctulata

XML Treatment for Atrichelaphinis (Heterelaphinis)

XML Treatment for Atrichelaphinis (Heterelaphinis) quadripunctata

XML Treatment for Atrichelaphinis (Heterelaphinis) sexualis

XML Treatment for Atrichelaphinis (Heterelaphinis) nigra

XML Treatment for Atrichelaphinis (Eugeaphinis)

XML Treatment for Atrichelaphinis (Eugeaphinis) deplanata deplanata

XML Treatment for Atrichelaphinis (Eugeaphinis) deplanata minettii

XML Treatment for Atrichelaphinis (Eugeaphinis) rhodesiana

XML Treatment for Atrichelaphinis (Eugeaphinis) bomboesbergica

XML Treatment for Atrichelaphinis (Eugeaphinis) garnieri

XML Treatment for Atrichelaphinis (Eugeaphinis) simillima

XML Treatment for Atrichelaphinis (Eugeaphinis) sternalis

XML Treatment for Atrichelaphinis (Eugeaphinis) vermiculata

XML Treatment for Atrichelaphinis (Eugeaphinis) bjornstadi
